# Synthesis of Trifluoromethylated Monoterpene Amino Alcohols

**DOI:** 10.3390/molecules27207068

**Published:** 2022-10-20

**Authors:** Polina A. Petrova, Denis V. Sudarikov, Larisa L. Frolova, Roman V. Rumyantcev, Svetlana A. Rubtsova, Aleksandr V. Kutchin

**Affiliations:** 1 Institute of Chemistry, FRC “Komi Scientific Centre”, Ural Branch of the Russian Academy of Sciences Pervomayskaya St. 48, 167000 Syktyvkar, Russia; 2 Biologically Active Terpenoids Laboratory, Kazan Federal University, 18 Kremlevskaya Street, 420008 Kazan, Russia; 3 G.A. Razuvaev Institute of Organometallic Chemistry of Russian Academy of Sciences, 49 Tropinina St., 603950 Nizhny Novgorod, Russia

**Keywords:** trifluoromethylation, Ruppert–Prakash reagent, chiral amino alcohol, pinane, bornane, monoterpenoids

## Abstract

For the first time, monoterpene trifluoromethylated β-hydroxy-benzyl-*O*-oximes were synthesized in 81–95% yields by nucleophilic addition of the Ruppert–Prakash reagent (TMSCF_3_) to the corresponding β-keto-benzyl-*O*-oximes based on (+)-nopinone, (−)-verbanone and (+)-camphoroquinone. Trifluoromethylation has been determined to entirely proceed chemo- and stereoselective at the C=O rather than C=N bond. Trifluoromethylated benzyl-*O*-oximes were reduced to the corresponding α-trifluoromethyl-β-amino alcohols in 82–88% yields. The structure and configuration of the compounds obtained have been established.

## 1. Introduction

Derivatives of monoterpenoids have a wide spectrum of antimicrobial activity against certain pathogenic species of bacteria and fungi [1]. The binding site of cyclic terpene hydrocarbons is located in the cell membrane of pathogenic microorganisms [2]. Many monoterpenoids, such as α- and β-pinenes, γ-terpinene, limonene, are capable of inhibiting respiration and other energy-dependent processes localized in fungal cell membranes [3].

It is known that the introduction of fluorine-containing groups into the molecule of a substance leads to an increase in membrane permeability, as well as an increase in resistance to biodegradation in comparison with their non-fluorinated analogues [4,5]. For this reason, about 25% of all modern pharmaceuticals contain fluorine atoms [6,7,8]. These transformations can lead to a change in the biological activity of the resulting compounds, as well as a new way of substrate–receptor interactions in comparison with hydrocarbon analogues [9,10,11,12].

Natural asymmetric molecules are a good starting point for the synthesis of chiral compounds because they are usually enantiomerically pure, obtained from renewable sources, and in most cases inexpensive. Terpenes are excellent natural asymmetric building blocks: they are mainly produced by various plants, some of them can be converted into more complex compounds used, for example, as ligands or catalysts for asymmetric reactions [13].

It is known that chiral β-amino alcohols [14,15], including terpene ones [16,17,18,19], are organocatalysts for a wide range of reactions of asymmetric synthesis, such as Diels–Alder cycloaddition, 1,3-dipolar cycloaddition, aldol condensation, Michael addition, cascade cyclization, Morita–Baylis–Hillman reaction, Friedel–Kraftz alkylation of indoles, allylation of isatins, and epoxidation of olefins.

It has been shown that the introduction of fluoroalkyl groups, including the trifluoromethyl group, into many chiral ligands, chiral auxiliaries, and chiral substrates improved their ability to induce asymmetry in stereoselective reactions [20,21,22,23]. Chiral α-trifluoromethyl-β-amino alcohols improved the stereoselectivity of addition reactions of diethylzinc and the Reformatsky reagent to carbonyl compounds and imines compared to their non-fluorinated analogs [22,24]. The authors attribute the effect of increasing stereoselectivity and reaction rate to strong electron-withdrawing properties, a large steric effect, as well as electrostatic repulsion between the local negative charge of the trifluoromethyl group and the charge of attacking nucleophiles [22].

In addition, fluorine-containing compounds are easily identified by ^19^F NMR spectroscopy and related homo- and heterocorrelation techniques due to the fact that the nucleus of the ^19^F fluorine atom has a spin ½, with an unprecedented natural abundance (100%) and a relatively high gyromagnetic ratio (83% of γ^1^H), which results in a strong signal. The large range of chemical shifts observed for fluorine nuclei means that ^19^F NMR spectroscopy is a very sensitive source of changes in the electronic environment and changes in the local dielectric medium [25,26]. These advantages, as well as the absence of background noise and the considerable simplicity of ^19^F NMR spectra compared to ^13^C, ^1^H, ^15^N nuclei, make it possible to study fluorine-containing compounds in biological media [25,27,28], to study the mechanisms and kinetics of reactions [29,30,31], including catalytic reactions [32,33]. Chiral fluorine-containing derivatizing agents make it easy to evaluate the enantiomeric purity of amines and amino alcohols by ^19^F NMR [34,35,36,37,38].

Based on the foregoing, the synthesis of chiral trifluoromethylated amino alcohols based on natural monoterpenoids is of undoubted interest. In this work, based on verbanone, nopinone, and camphorquinone, we synthesized the corresponding ketooximes, benzyl-*O*-oximes, trifluoromethylated benzyl-*O*-oxymoalcohols, and trifluoromethylated amino alcohols.

## 2. Results

**Scheme 1:**
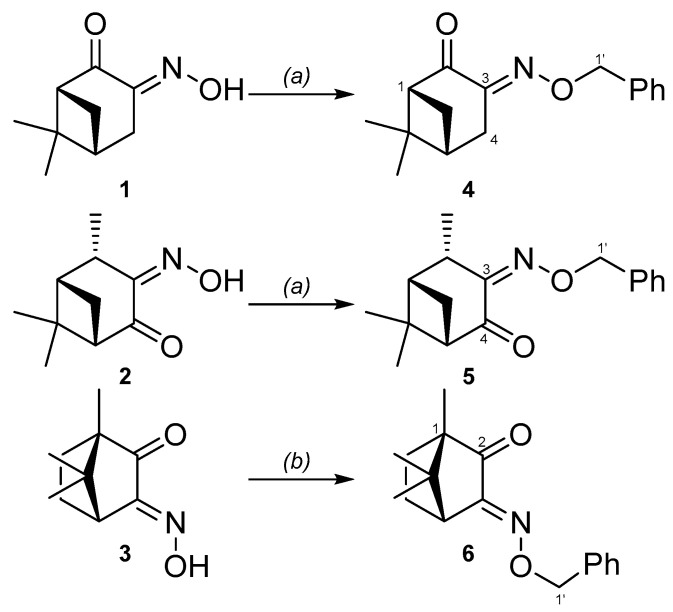
Synthesis of benzyl-*O*-oximes **4**–**6** from oximes **1**–**3**. Reagents and conditions: (**a**) Cs_2_CO_3_, BnCl, MeCN, rt, 48 h. (**b**) t-BuOK, BnCl, THF, 4 °C → rt, 24 h.

For this study, we used ketooximes **1**–**3** based on (+)-nopinone [39], (−)-verbanone [18], and (+)-camphoroquinone [40], which were synthesized according to already known methods. The corresponding benzyl-*O*-oximes **4**–**6** were obtained from the resulting oximes **1**–**3** in 68, 55, 45% yields, respectively (Scheme 1). The benzyl group was previously introduced to protect the OH group of the oxime before trifluoromethylation of the obtained compounds.

The IR spectra of compounds **4**–**6** contain absorption bands characteristic of the carbonyl group in the region of 1715–1717 cm^−1^, characteristic of the C=N–O group in the region of 1584–1684 cm^−1^. The ^1^H NMR spectra contain the signals of the protons of the methylene group C-1′ at 5.37 ppm for compound **4**, at 5.4 ppm for **5**; at 5.28 ppm for **6** and a multiplet of the phenyl fragment in the range 7.31–7.41 ppm for **4**–**6**. In the ^13^C NMR spectra, the signals of these functional groups are present at 77.9 ppm for **4**, at 78 ppm for **5**, at 77.3 ppm for **6** and the multiplet of the phenyl fragment in the regions 128.2–128.4 ppm for **4**–**6**, respectively.

Significantly, oximes **4**–**6** have two reaction centers C=O and C=N bonds, and both of them can be subjected to trifluoromethylation, for example, as demonstrated in [41,42,43,44] for imines and sulfinimines, which react at the C=N bond, and for monoterpene ketooximes **4**–**6** undergoing trifluoromethylation to yield the products solely at the C=O bond.

**Scheme 2:**
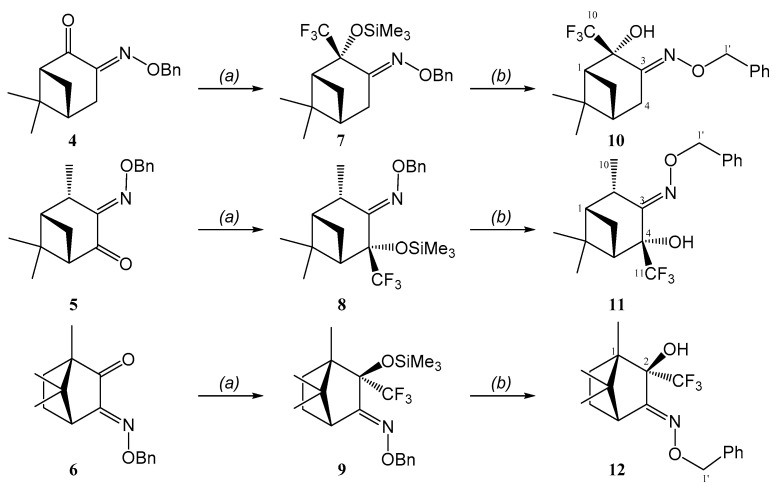
Synthesis of β-trifluoromethyl-β-hydroxy-benzyl-*O*-oximes **10**–**12** from β-keto-benzyl-*O*-oximes **4**–**6**. Reagents and conditions: (**a**) TMSCF_3_, CsF, THF, 4 °C → rt, 4 h. (**b**) TBAF·3H_2_O, THF, rt, 30 min.

Nucleophilic addition of the Ruppert–Prakash reagent–trifluoromethyltrimethylsilane (TMSCF_3_) [45] to β-keto-benzyl-*O*-oximes **4**–**6** at the double C=O bond is carried out in THF at 4 °C in an argon atmosphere in the presence of an initiator—cesium fluoride (CsF). At the first stage, trimethylsilyl ethers **7**–**9** are formed, which, after addition of tetrabutylammonium fluoride hydrate (TBAF·3H_2_O), form new trifluoromethyl alcohols **10**–**12** in 81, 89 and 95% yields, respectively (Scheme 2).

The IR spectra of compounds **10**–**12** contain absorption bands characteristic of the hydroxyl group in the region 3437–3560 cm^−1^, characteristic of the C=N–O group in the region 1618–1688 cm^−1^, absorption bands corresponding to the CF_3_ group at 1271, 1171, and 1096 cm^−1^ for **10**; at 1283, 1169, 1105 cm^−1^ for **11**; at 1265, 1180, and 1103 cm^−1^ for **12**.

The ^1^H NMR spectra of compounds **10**–**12** contain singlets of the proton of the hydroxyl group at 3.27 ppm for **10**; 3.20 ppm for **11**; 2.76 for **12** and a multiplet of the phenyl fragment in the range of 7.31–7.41 ppm for **10**–**12**. The ^13^C NMR spectra show quartets of the C-2 carbon atom at 77.9 ppm (*J*_F_ 27.6 Hz) for **10**; at 78.9 ppm (*J*_F_ 27.6 Hz) for **12**. There is a quartet of C-4 carbon at 78.6 ppm (*J*_F_ 26.5 Hz) in the ^13^C NMR spectrum of compound **11**. The quartet of the C-10 carbon of compound **10** is present at 125.0 ppm (*J*_F_ 288.6 Hz). The quartets of the C-11 carbon atom of compounds **11**, **12** are present at 125.2 ppm (*J*_F_ 288.6 Hz) for **11**; at 125.2 ppm (*J*_F_ 287.5 Hz) for **12**. Singlets of the trifluoromethyl group of compounds **10**–**12** appear in the ^19^F NMR spectra in the range from −71.8 to −74.6 ppm.

For each of the benzyl-*O*-oximes **4**–**6** in the trifluoromethylation reaction, only (2*S*)-**10**, (4*R*)-**11**, (2*R*)-**12** diastereomers are formed from two theoretically possible diastereomers in 81, 89 and 95% yields, respectively (Scheme 2).

**Figure 1:**
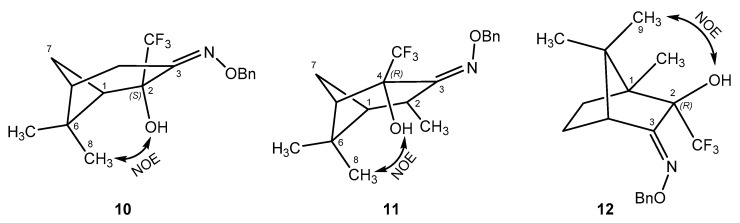
Structure and *NOE* interactions of compounds (2*S*)-**10**, (4*R*)-**11**, (2*R*)-**12**.

The configuration of C-2, C-4 and C-2 atoms of compounds **10**–**12**, respectively, was established by ^1^H NMR NOESY spectroscopy by the presence of *NOE* interactions between the protons of the hydroxyl group and the C-8 methyl group in compounds **10** and **11**, between the protons of the hydroxyl group and the C-9 methyl group of compound **12** (Figure 1).

**Scheme 3:**
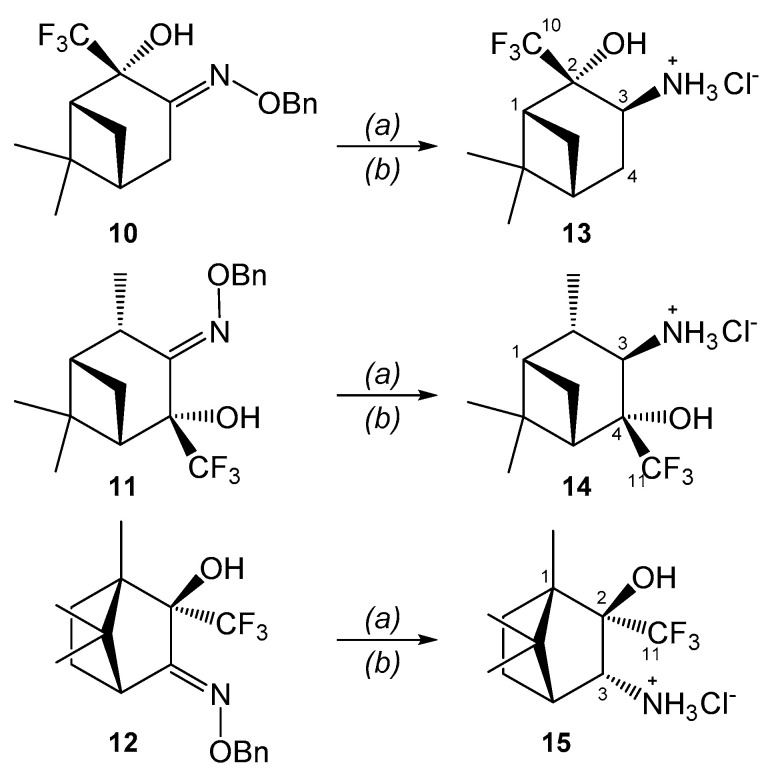
Synthesis of hydrochlorides of trifluoromethylated amino alcohols **13**–**15** from trifluoromethylated benzyl-*O*-oximes **10**–**12**. Reagents and conditions: (**a**) LiAlH_4_, Et_2_O, 4 °C → rt, 12 h. (**b**) HCl (gas), Et_2_O, 4 °C.

Trifluoromethylated benzyl-*O*-oximes **10**–**12** based on (+)-nopinone, (−)-verbanone and (+)-camphoroquinone were reduced with LiAlH_4_ to the corresponding amines isolated as hydrochlorides **13**–**15** in 81–95% yields (Scheme 3).

The IR spectra of compounds **13**–**15** contain absorption bands characteristic of the hydroxyl group in the region of 3298–3558 cm^−1^, characteristic of the NH_3_^+^ group in the region of 2922–3080 cm^−1^, absorption bands corresponding to the CF_3_ group in the regions of 1128–1198 cm^−1^ for **13**, 1126–1194 cm^−1^ for **14**, and 1121–1186 cm^−1^ for **15**.

The ^1^H NMR spectra of compounds **13**–**15** contain singlets of the protons of the OH and NH_3_^+^ groups at 4.75 ppm and there are no signals of the phenyl fragment compared to the original substrates. The ^13^C NMR spectra of compounds **13** and **15** contain quartets of the C-2 carbon atom at 78.2 ppm (*J*_F_ 25.4 Hz) for **13**; at 80.6 ppm (*J*_F_ 26.5 Hz) for 15. There is a quartet of the C-4 carbon atom at 78.1 ppm (*J*_F_ 26.5 Hz) in the ^13^C NMR spectrum of compound **14**. The quartet of the trifluoromethyl group C-10 of compound **13** is at 125.3 ppm (*J*_F_ 288.6 Hz). Quartets of the trifluoromethyl groups C-11 of compounds **14**, **15** are present at 125.3 ppm (*J*_F_ 287.5 Hz) for **14**; at 125.4 ppm (*J*_F_ 288.6 Hz) for **15**, respectively. There are singlets in the range from −72.6 to −68.2 ppm in the ^19^F NMR spectra of **13**–**15**.

**Figure 2:**
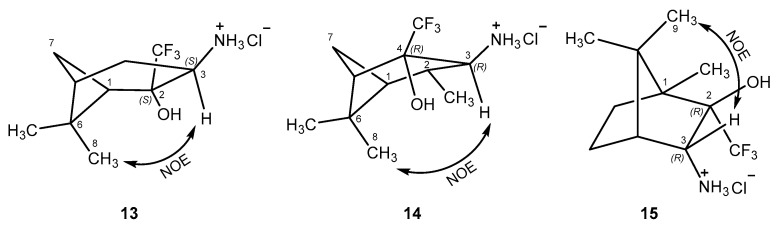
Structure and *NOE* interactions of compounds (2*S*,3*S*)-**13**, (3*R*,4*R*)-**14**, (2*R*,3*R*)-**15**.

The configuration of the C-3 atom of compounds **13**–**15** was established by ^1^H NOESY NMR spectroscopy by the presence of NOE interactions between the H-3 protons and the methyl group C-8 in **13** and **14**, between the H-3 protons and the methyl group C-9 in the compound **15** (Figure 2).

**Figure 3:**
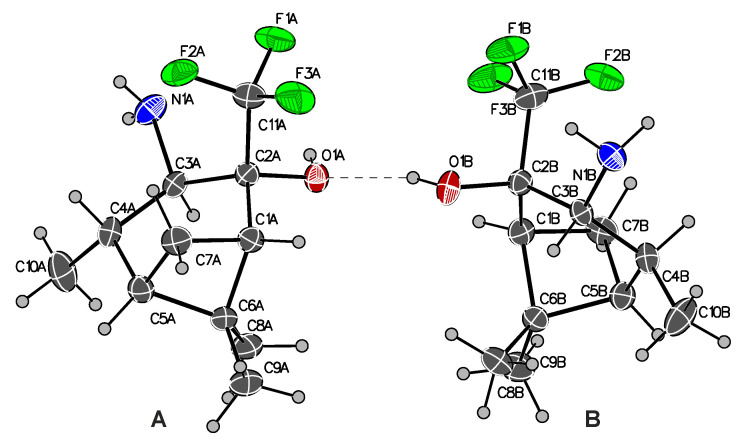
Molecular structure of two independent molecules (**A**,**B**) of compound **16** with thermal ellipsoids drawn at the 30% probability level.

A single crystal of free amine **16** was obtained after alkaline extraction of hydrochloride **14** with Et_2_O. The configuration of free amine **16** was confirmed by X-ray diffraction analysis (Figure 3). This compound crystallizes in the chiral space group P2_1_2_1_2_1_ of the orthorhombic system. There are two independent molecules (**A** and **B**) of **16** in the asymmetric unit cell. They have the same molecular structure. The root–mean–square deviation of atomic positions of **A** and **B** molecules is 0.056 Å. The carbon atoms C(1), C(2), C(3), C(4), and C(5) lie almost in the same plane. The average deviation of atoms from the plane is 0.085 Å. The trifluoromethyl group, the amino group, and the methylene group are on the same side of this plane. The main geometric characteristics in **16** are in good agreement with related carbocyclic compounds [18,46].

In a crystal, neighboring molecules are oriented in such a way that intermolecular hydrogen bonds are realized O-H...O (2.03 Å), O-H...N (2.07 Å), N-H...N (2.47 Å), and N-H...O (2.48 Å). As a result, endless molecular chains **A**-**B**-**A**-**B** are formed.

## 3. Materials and Methods

### 3.1. General Information

FT-IR spectra were recorded on a Shimadzu IR Prestige 21 on thin films or KBr pellets; *ν* in cm^−1^. ^1^H and ^13^C NMR spectra were registered on a Bruker Avance 300 spectrometer (300.17 MHz for ^1^H, 75.48 MHz for ^13^C and 282.44 MHz for ^19^F) in CDCl_3_, *J* in Hz (See Supplementary Materials). The signals were assigned using COSY, NOESY, HSQC, HMBC techniques, and ^13^C NMR spectra in *J*-modulation mode. Automatic analyzer EA 1110 CHNS-O was employed for elemental analysis. The melting points were measured on a Sanyo Gallenkamp MPD350.BM3.5 and were not corrected. Optical rotations were performed with automatized digital polarimeter Optical Activity PolAAr 3001. Thin layer chromatography (TLC) was performed on Sorbfil plates; spots were visualized by treatment with 10% phosphomolybdic acid in ethanol, 5% vanillin and 0.005% H_2_SO_4_ in ethanol, 5% KMnO_4_, and 0.005% H_2_SO_4_ in H_2_O. Silica gel 60 (70–230 mesh, Alfa Aesar, Lancashire, UK) was used for column chromatography (CC). For both TLC and CC the same eluent systems were used.

*X-ray Data Collection and Structure Refinement.* The diffraction data for compound **16** were collected on a Bruker D8 Quest diffractometer (Mo-K_α_ radiation, ω-scan technique, λ = 0.71073 Å) at 298(2) K. The intensity data were integrated by the SAINT [47] program. The structure was solved by dual methods [48] and was refined on $F_{\mathrm{hkl}}^{2}$ using the SHELXTL package [49]. The SADABS program [50] was used to perform absorption corrections. All non-hydrogen atoms were refined anisotropically. All H-atoms, with the exception of hydrogens of the hydroxyl and amino groups, were placed in calculated positions and were refined using a riding model (U_iso_(H) = 1.5U_eq_(C) for CH_3_ groups and U_iso_(H) = 1.2U_eq_(C) for other groups). The H(1)-H(6) atoms in **16** were located from the differential Fourier map and were refined isotropically. CCDC 2191818 contains the supplementary crystallographic data accessed on 19 October 2022. These data can be obtained free of charge from The Cambridge Crystallographic Data Centre via https://www.ccdc.cam.ac.uk/structures.

Commercially available reagents such as (trifluoromethyl)trimethylsilane TMSCF_3_ (purity 98%, Alfa Aesar, Lancashire, UK), caesium fluoride CsF (purity 98%, Alfa Aesar, Lancashire, UK), tetra-*N*-butylammonium fluoride trihydrate TBAF·3H_2_O (purity 98%, Alfa Aesar, Lancashire, UK), lithium aluminum hydride LiAlH_4_ (purity 95%, Sigma-Aldrich, St. Louis, USA) were used directly as supplied without further purification. All solvents used for the reactions were distilled. (1*R*,5*R*,*E*)-3-(Hydroxyimino)-6,6-dimethylbicyclo[3.1.1]heptan-2-one (**1**), mp 180 °C, [39], (1*S*,4*S*,5*S*,*Z*)-3-(hydroxyimino)-4,6,6-trimethylbicyclo[3.1.1]heptan-2-one (**2**), mp 135 °C, [18], and (1*S*,4*R*,*E*)-3-(hydroxyimino)-1,7,7-trimethylbicyclo[2.2.1]heptan-2-one (**3**), mp 118 °C, [40] were synthesized in accordance with known methods.

### 3.2. General Procedure for the Synthesis of Benzyl-O-Oximes ***4*** and ***5***

In a two-necked flask equipped with a stirrer and a reflux condenser, oxime **1** or **2** (2.63 mmol) in 15 mL of acetonitrile was placed under argon. Cs_2_CO_3_ (5.27 mmol) was then added, and benzyl chloride (5.27 mmol) was added dropwise after 5 min of stirring. The resulting mixture was stirred for 3 h at room temperature. The reaction progress was monitored by TLC (eluent, chloroform). The solvent was distilled off under vacuum, H_2_O (30 mL) was added to the residue, extracted with Et_2_O, the organic layer was washed with brine and dried over Na_2_SO_4_. The solvent was distilled off under reduced pressure. The reaction products were isolated by silica gel column chromatography.

*(1R,5R,E)-3-((Benzyloxy)imino)-6,6-dimethylbicyclo[3.1.1]heptan-2-one* (**4**). Yield: 68%; light brown oil; ${\left[\alpha \right]}_{D}^{25}=$+19.70 (*c* = 0.99 in CHCl_3_); R*_f_* 0.38 (petr. ether/EtOAc, 3:1); ^1^H NMR (CDCl_3_, δ, ppm, *J*/Hz): 0.91 (s, 3H, H^8^), 1.38 (s, 3H, C^9^H_3_), 1.53 (d, 1H, *J =* 11.0, H^7^^α^), 2.25–2.32 (m, 1H, H^5^), 2.65–2.88 (m, 4H, H^1^, H^7^^β^, H^4^^α^, H^4^^β^), 5.37 (s, 2H, H^1′^^α^, H^1′^^β^), 7.32–7.41 (m, 5H, H^3′^, H^4′^, H^5′^, H^6′^, H^7′^); ^13^C NMR (CDCl_3_, δ, ppm): 21.5 (C^8^), 26.2 (C^9^), 28.0 (C^7^), 28.6 (C^4^), 37.6 (C^5^), 41.7 (C^6^), 56.6 (C^1^), 77.9 (C^1′^), 128.2 (C^5′^), 128.3 (C^4′,6′^), 128.4 (C^3′,7′^), 136.6 (C^2′^), 152.7 (C^3^), 198.2 (C^2^); IR spectrum (KBr, ν, cm^−1^): 1715 (C=O), 1684 (C=N−O); elemental analysis calcd (%) for C_16_H_19_NO_2_: C 74.68, H 7.44, N 5.44; found: C 74.08, H 7.26, N 5.14.

*(1S,4S,5S,E)-3-((Benzyloxy)imino)-4,6,6-trimethylbicyclo[3.1.1]heptan-2-one* (**5**). Yield: 55%; light brown oil; ${\left[\alpha \right]}_{D}^{25}=$−1.2 (*c* = 1.0 in CHCl_3_); R*_f_* 0.29 (CHCl_3_/petr. ether, 2:1); ^1^H NMR (CDCl_3_, δ, ppm, *J*/Hz): 1.05 (s, 3H, H^8^), 1.37 (d, *J =* 7.2, 3H, H^10^), 1.39 (s, 3H, C^9^H_3_), 1.45 (d, 1H, *J =* 11.0, H^7^^α^), 2.17 (td, 1H, *J =* 5.8, 3.0, H^1^), 2.66 (ddd, 1H, *J =* 11.0, 6.1, 5.8, H^7^^β^), 2.73 (t, 1H, *J =* 5.8, H^5^), 3.18 (qd, 1H, *J =* 7.1, 3.0, H^2^), 5.32–5.42 (m, 2H, H^1′^^α^, H^1′^^β^), 7.31–7.41 (m, 5H, H^3′^, H^4′^, H^5′^, H^6′^, H^7′^); ^13^C NMR (CDCl_3_, δ, ppm): 16.5 (C^10^), 23.9 (C^8^), 27.3 (C^9^), 28.2 (C^7^), 36.9 (C^2^), 42.3 (C^6^), 45.7 (C^1^), 56.6 (C^5^), 78.0 (C^1′^), 128.1 (C^5′^), 128.2 (C^4′,6′^), 128.4 (C^3′,7′^), 136.6 (C^2′^), 156.3 (C^3^), 199.0 (C^4^); IR spectrum (KBr, ν, cm^−1^): 1717 (C=O), 1584 (C=N−O); elemental analysis calcd (%) for C_17_H_21_NO_2_: C 75.25, H 7.80, N 5.16; found: C 75.05, H 7.52, N 5.01.

*(1S,4R,E)-3-((Benzyloxy)imino)-1,7,7-trimethylbicyclo[2.2.1]heptan-2-one* (**6**). In a two-necked flask equipped with a stirrer and a reflux condenser, (+)-camphorquinone oxime **3** (0.59 mmol) in 3 mL of THF (dry) was placed under argon. After ice-bath cooling the mixture, t-BuOK (0.65 mmol) was added and the flask was purged with argon. Benzyl chloride (2.06 mmol) was added after 20 min of stirring. The resulting mixture was stirred overnight at room temperature. The progress of the reaction was monitored by TLC (eluent, petr.ether:EtOAc, 10:1). At the end of the reaction, H_2_O (20 mL) was added, the reaction mixture was extracted with diethyl ether, the organic layer was washed with brine and dried over Na_2_SO_4_. The solvent was distilled off under reduced pressure. The reaction product was isolated by silica gel column chromatography. Yield: 45%; light brown oil; ${\left[\alpha \right]}_{D}^{25}=$−134.4 (*c* = 1.15 in CHCl_3_); R*_f_* 0.23 (petr. ether/Et_2_O, 5:1); ^1^H NMR (CDCl_3_, δ, ppm, *J*/Hz): 0.88 (s, 3H, H^8^), 0.98 (s, 3H, C^9^H_3_), 1.03 (s, 3H, C^10^H_3_), 1.47–1.62 (m, 2H, H^5^^α^, H^6^^α^), 1.71–1.83 (m, 1H, H^6^^β^), 1.96–2.06 (m, 1H, H^5^^β^), 3.21 (d, 1H, *J =* 4.4, H^4^), 5.28 (s, 2H, H^1′^^α^, H^1′^^β^), 7.33–7.37 (m, 5H, H^3′^, H^4′^, H^5′^, H^6′^, H^7′^); ^13^C NMR (CDCl_3_, δ, ppm): 9.0 (C^10^), 17.6 (C^9^), 20.6 (C^8^), 23.9 (C^5^), 30.7 (C^6^), 44.8 (C^7^), 47.4 (C^4^), 58.5 (C^1^), 77.3 (C^1′^), 128.0 (C^5′^), 128.1 (C^4′,6′^), 128.4 (C^3′,7′^), 137.0 (C^2′^), 159.3 (C^3^), 203.9 (C^2^); IR spectrum (KBr, ν, cm^−1^): 1715 (C=O), 1634 (C=N−O); elemental analysis calcd (%) for C_17_H_21_NO_2_: C 75.25, H 7.80, N 5.16; found: C 75.10, H 7.56, N 5.09.

### 3.3. General Procedure for Trifluoromethylation of β-Keto-Benzyl-O-Oximes ***4***–***6***

In a two-necked flask equipped with a stirrer and reflux condenser, cooled in an ice bath under argon, benzyl-*O*-oxime **4** (or **5**, **6**, 1.36 mmol) was placed in 6 mL of THF (dry). After cooling the mixture, CsF (0.68 mmol) and TMSCF_3_ (4.08 mmol) were added with stirring. The resulting mixture was stirred for 4 h (control by TLC until the disappearance of the substrate). After that, the ice-bath was removed and TBAF·3H_2_O (1.36 mmol) was added. The progress of the reaction was monitored by TLC (eluent, pet.ether:EtOAc, 3:1). A saturated solution of NH_4_Cl (20 mL) was added, the reaction products were extracted with diethyl ether, the organic layer was washed with brine and dried over Na_2_SO_4_. The solvent was distilled off under vacuum. The reaction products were isolated by column chromatography.

*((1R,2S,5R,E)-2-Hydroxy-6,6-dimethyl-2-(trifluoromethyl)bicyclo[3.1.1]heptan-3-one O-benzyl oxime* (**10**). Yield: 81%; light brown oil; ${\left[\alpha \right]}_{D}^{25}=$−9.69 (*c* = 0.98 in CHCl_3_); R*_f_* 0.39 (petr. ether/EtOAc, 10:1); ^1^H NMR (CDCl_3_, δ, ppm, *J*/Hz): 0.95 (s, 3H, H^8^), 1.35 (s, 3H, C^9^H_3_), 1.53 (d, 1H, *J =* 11.3, H^7^^α^), 1.99–2.04 (m, 1H, H^5^), 2.34 (t, 1H, *J =* 5.9, H^1^), 2.40–2.54 (m, 2H, H^7^^β^, H^4^^α^), 3.02 (dt, 1H, *J =* 18.7, 3.0, H^4^^β^), 3.27 (s, 1H, OH), 5.16–5.25 (m, 2H, H^1′^^α^, H^1′^^β^), 7.34–7.39 (m, 5H, H^3′^, H^4′^, H^5′^, H^6′^, H^7′^); ^13^C NMR (CDCl_3_, δ, ppm): 21.9 (C^8^), 26.7 (C^7^), 26.8 (C^9^), 30.2 (C^4^), 37.4 (C^5^), 40.0 (C^6^), 45.2 (C^1^), 76.7 (C^1′^), 77.9 (q, *J*_F_ = 27.6, C^2^), 125.0 (q, *J*_F_ = 288.6, C^10^), 128.0 (C^5′,4′,6′^), 128.4 (C^3′,7′^), 137.4 (C^2′^), 156.7 (C^3^); ^19^F NMR (CDCl_3_, δ, ppm, *J*/Hz): –74.6 (s, 3F, C^11^F_3_); IR spectrum (KBr, ν, cm^−1^): 3560 (OH), 1627 (C=N−O), 1271, 1171, 1096 (CF_3_); elemental analysis calcd (%) for C_17_H_20_F_3_NO_2_: C 62.38, H 6.16, N 4.28; found: C 62.01, H 6.12, N 4.17.

*(1S,2R,4S,5S,E)-2-Hydroxy-4,6,6-trimethyl-2-(trifluoromethyl)bicyclo[3.1.1]heptan-3-one O-benzyl oxime* (**11**). Yield: 89%; light brown oil; ${\left[\alpha \right]}_{D}^{26}=$+17.9 (*c* = 1.0 in CHCl_3_); R*_f_* 0.20 (petr. ether/CH_2_Cl_2_, 2:1); ^1^H NMR (CDCl_3_, δ, ppm, *J*/Hz): 1.11 (s, 3H, H^8^), 1.35 (s, 3H, C^9^H_3_), 1.41 (d, 1H, *J =* 11.6, H^7^^α^), 1.47 (d, *J =* 7.2, 3H, H^10^), 1.85 (td, 1H, *J =* 5.8, 2.0, H^1^), 2.33 (t, 1H, *J =* 5.8, H^5^), 2.42 (dt, 1H, *J =* 11.6, 6.1, H^7^^β^), 2.92 (qd, 1H, *J =* 7.1, 2.0, H^2^), 3.20 (s, 1H, OH), 5.13–5.22 (m, 2H, H^1′^^α^, H^1′^^β^), 7.32–7.40 (m, 5H, H^3′^, H^4′^, H^5′^, H^6′^, H^7′^); ^13^C NMR (CDCl_3_, δ, ppm): 19.1 (C^10^), 24.2 (C^8^), 27.0 (C^7^), 27.9 (C^9^), 39.2 (C^2^), 39.7 (C^6^), 44.9 (C^5^), 46.7 (C^1^), 77.0 (C^1′^), 78.6 (q, *J*_F_ = 26.5, C^4^), 125.2 (q, *J*_F_ = 288.6, C^11^), 127.9 (C^5′^), 128.0 (C^4′,6′^), 128.4 (C^3′,7′^), 137.4 (C^2′^), 158.5 (C^3^); ^19^F NMR (CDCl_3_, δ, ppm, *J*/Hz): –74.2 (s, 3F, C^11^F_3_); IR spectrum (KBr, ν, cm^−1^): 3557 (OH), 1618 (C=N−O), 1265, 1180, 1103 (CF_3_); elemental analysis calcd (%) for C_18_H_22_F_3_NO_2_: C 63.33, H 6.50, N 4.10; found: C 63.13, H 6.34, N 4.32.

*(1R,3R,4S,E)-3-Hydroxy-4,7,7-trimethyl-3-(trifluoromethyl)bicyclo[2.2.1]heptan-2-one O-benzyl oxime* (**12**). Yield: 95%; light brown oil; ${\left[\alpha \right]}_{D}^{25}=$−37.9 (*c* = 0.82 in CHCl_3_); R*_f_* 0.37 (petr. ether/Et_2_O, 10:1); ^1^H NMR (CDCl_3_, δ, ppm, *J*/Hz): 0.94 (s, 3H, H^8^), 1.04 (s, 3H, C^9^H_3_), 1.07 (s, 3H, C^10^H_3_), 1.36–1.45 (m, 1H, H^5^^α^), 1.58–1.89 (m, 3H, H^6^^α^, H^6^^β^, H^5^^β^), 2.76 (s, 1H, OH), 3.12 (d, 1H, *J =* 4.4, H^4^), 5.09–5.18 (m, 2H, H^1′^^α^, H^1′^^β^), 7.30–7.39 (m, 5H, H^3′^, H^4′^, H^5′^, H^6′^, H^7′^); ^13^C NMR (CDCl_3_, δ, ppm): 9.8 (C^10^), 18.7 (C^9^), 21.9 (C^8^), 22.3 (C^5^), 28.9 (C^6^), 48.0 (C^7^), 48.3 (C^4^), 52.6 (C^1^), 76.2 (C^1′^), 78.9 (q, *J*_F_ = 27.6, C^2^), 125.2 (q, *J*_F_ = 287.5, C^11^), 127.8 (C^5′^), 127.9 (C^4′,6′^), 128.3 (C^3′,7′^), 137.8 (C^2′^), 164.7 (C^3^); ^19^F NMR (CDCl_3_, δ, ppm, *J*/Hz): –71.8 (s, 3F, C^11^F_3_); IR spectrum (KBr, ν, cm^−1^): 3437 (OH), 1688 (C=N−O), 1283, 1169, 1105 (CF_3_); elemental analysis calcd (%) for C_18_H_22_F_3_NO_2_: C 63.33, H 6.50, N 4.10; found: C 63.71, H 6.62, N 4.38.

### 3.4. General Procedure for the Preparation of Trifluoromethylated Amino Alcohols ***13***–***15***

Trifluoromethylated β-trifluoromethyl-β-hydroxy-benzyl-*O*-oxime **10** (or **11**, **12**, 0.54 mmol) in 15 mL of dry Et_2_O was placed into a two-necked flask equipped with a stirrer, cooled to 4 °C in an argon atmosphere. LiAlH_4_ (1.65 mmol) was added in portions with stirring. The reaction mixture was stirred at room temperature for a day, the progress of the reaction was monitored by TLC (eluent, pet.ether: EtOAc, 10:1). The mixture was cooled again in an ice bath, then Et_2_O (20 mL) was added and 5% KOH solution was carefully poured until phase separation and the aqueous layer was extracted with Et_2_O. The combined organic phases were washed with brine and dried over Na_2_SO_4_. The solvent was distilled off in vacuo. The resulting amines were isolated in the hydrochlorides form in Et_2_O solution by blowing dry HCl into the flask until the precipitation ceased. The hydrochlorides were purified by washing with a mixture of hexane- Et_2_O (1:1).

*(1R,2S,3S,5R)-2-Hydroxy-6,6-dimethyl-2-(trifluoromethyl)bicyclo[3.1.1]heptan-3-ammonium chloride* (**13**). Yield: 88%; white powder; mp = 218 °C (decomposition); ${\left[\alpha \right]}_{D}^{27}=$+33.3 (*c* = 0.74 in MeOH); ^1^H NMR (D_2_O, δ, ppm, *J*/Hz): 1.04 (s, 3H, H^8^), 1.30 (s, 3H, C^9^H_3_), 1.36 (d, 1H, *J =* 11.6, H^7^^α^), 1.78 (ddd, 1H, *J =* 14.1, 6.4, 1.5, H^4^^β^), 2.05–2.12 (m, 1H, H^5^), 2.37 (t, 1H, *J =* 5.8, H^1^), 2.40–2.49 (m, 1H, H^7^^β^), 2.68 (ddt, 1H, *J =* 13.8, 11.0, 3.2, H^4^^β^); 4.04 (dd, 1H, *J =* 10.3, 6.2, H^3^), 4.75 (s, 4H, OH, NH_3_^+^); ^13^C NMR (D_2_O, δ, ppm): 22.6 (C^8^), 26.0 (C^7^), 26.5 (C^9^), 31.9 (C^4^), 38.6 (C^6^), 39.0 (C^5^), 47.2 (C^1^), 51.3 (C^3^), 78.2 (q, *J*_F_ = 25.4, C^2^), 125.3 (q, *J*_F_ = 288.6, C^10^); ^19^F NMR (D_2_O, δ, ppm, *J*/Hz): –72.6 (s, 3F, C^10^F_3_); IR spectrum (KBr, ν, cm^−1^): 3558 (OH), 2959 (NH_3_^+^), 1601 (N), 1198, 1148, 1128 (CF_3_); elemental analysis calcd (%) for C_10_H_17_ClF_3_NO: C 46.25, H 6.60, N 5.39; found: C 46.61, H 6.82, N 5.51.

*(1S,2R,3R,4S,5S)-2-Hydroxy-4,6,6-trimethyl-2-(trifluoromethyl)bicyclo[3.1.1]heptan-3-ammonium chloride* (**14**). Yield: 89%; white powder; mp = 220 °C (decomposition); ${\left[\alpha \right]}_{D}^{25}=$−17.7 (*c* = 0.5 in MeOH); ^1^H NMR (D_2_O, δ, ppm, *J*/Hz): 1.15 (s, 3H, H^8^), 1.25 (d, *J =* 6.9, 3H, H^10^), 1.34 (s, 3H, C^9^H_3_), 1.34 (d, 1H, *J =* 11.3, H^7^^α^), 2.00 (t, 1H, *J =* 5.5, H^1^), 2.92 (quin, 1H, *J =* 7.5, H^2^), 2.39 (t, 1H, *J =* 5.8, H^5^), 2.49 (dt, 1H, *J =* 11.8, 6.1, H^7^^β^), 3.91 (d, 1H, *J =* 8.8, H^3^), 4.75 (s, 4H, OH, NH_3_^+^); ^13^C NMR (D_2_O, δ, ppm): 18.4 (C^10^), 23.9 (C^8^), 27.9 (C^7^), 27.9 (C^9^), 39.0 (C^6^), 40.1 (C^2^), 46.3 (C^1^), 48.5 (C^5^), 59.7 (C^3^), 78.1 (q, *J*_F_ = 26.5, C^4^), 125.3 (q, *J*_F_ = 287.5, C^11^); ^19^F NMR (D_2_O, δ, ppm, *J*/Hz): –71.1 (s, 3F, C^11^F_3_); IR spectrum (KBr, ν, cm^−1^): 3298 (OH), 2955, 2922 (NH_3_^+^), 1580 (N-H), 1194, 1150, 1126 (CF_3_); elemental analysis calcd (%) for C_11_H_19_ClF_3_NO: C 48.27, H 7.00, N 5.12; found: C 48.11, H 6.82, N 5.12. A single crystal of free amine **16** was obtained after alkaline extraction of hydrochloride **14** with Et_2_O. A colorless prismatic crystal of the orthorhombic system had size 0.56 × 0.47 × 0.42 mm, space group *P*2_1_2_1_2_1_, *a* = 7.8532(5), *b* = 15.7765(9), *c* = 19.0439(12) Å, *α* = *β* = *γ* = 90°, *V* = 2359.5(3) Å^3^, Z = 8, μ = 0.117 mm^−1^, *d*_calc_ = 1.336 g/cm^3^, F(000) = 1008. A dataset of 34,841 reflections was collected at scattering angles 2.139° < θ < 26.361°, of which 4820 were independent (R_int_ = 0.0287), including 4036 reflections with *I* > 2σ(*I*). The final refinement parameters were R_1_ = 0.0496, wR_2_ = 0.1013 (all data), R_1_ = 0.0376, wR_2_ = 0.0944 [*I* > 2σ(*I*)] with GooF = 1.062. Δρ*_e_* = 0.190/−0.169 *e* Å^–3^; Flack parameter = −0.16(15).

*(1R,2R,3R,4S)-3-Hydroxy-4,7,7-trimethyl-3-(trifluoromethyl)bicyclo[2.2.1]heptan-2-ammonium chloride* (**15**). Yield: 82%; white powder; mp = 222 °C (decomposition); ${\left[\alpha \right]}_{D}^{26}=$−11.9 (*c* = 0.8 in MeOH); ^1^H NMR (D_2_O δ, ppm, *J*/Hz): 0.95 (s, 3H, H^8^), 0.99 (s, 3H, C^10^H_3_), 1.14 (s, 3H, C^9^H_3_), 1.35–1.50 (m, 1H, H^5^^α^), 1.64–1.69 (m, 2H, H^6^^α^, H^6^^β^, H^5^^β^), 1.76–1.87 (m, 1H, H^5^^β^), 2.14 (t, 1H, *J =* 4.0, H^4^), 3.95–3.96 (m, 1H, H^3^), 4.75 (br.s, 4H, OH, NH_3_^+^); ^13^C NMR (D_2_O, δ, ppm): 10.3 (C^10^), 18.0 (C^5^), 19.3 (C^9^), 19.6 (C^8^), 27.9 (C^6^), 47.6 (C^4^), 48.0 (C^7^), 53.4 (C^1^), 60.7 (C^3^), 80.6 (q, *J*_F_ = 26.5, C^2^), 125.4 (q, *J*_F_ = 288.6, C^11^); ^19^F NMR (CDCl_3_, δ, ppm, *J*/Hz): –68.2 (s, 3F, C^11^F_3_); IR spectrum (KBr, ν, cm^−1^): 3339 (OH), 3080, 2964 (NH_3_^+^), 1585 (N-H), 1186, 1143, 1121 (CF_3_); elemental analysis calcd (%) for C_11_H_19_ClF_3_NO: C 48.27, H 7.00, N 5.12; found: C 48.61, H 7.12, N 5.34.

## 4. Conclusions

Thus, trifluoromethylated amino alcohols based on pinane and bornane monoterpenoids have been synthesized for the first time. The addition of the Ruppert–Prakash reagent to β-keto-benzyl-*O*-oximes, as well as the reduction of β-hydroxy-benzyl-*O*-oximes to the corresponding amino alcohols proceed stereoselectively with the formation of one of the diastereomers. Trifluoromethylation has been determined to entirely proceed at the C=O rather than C=N bond.

All compounds are isolated individually; the structure and configuration are proven by NMR and IR spectroscopy, elemental, and X-ray diffraction analysis. The obtained compounds may be of interest as biologically active substances and/or their precursors, as well as new chiral fluorine-containing auxiliaries, ligands or organocatalysts containing a trifluoromethyl group.

## Data Availability

Not applicable.

## Supplementary Materials

The following are available online at https://www.mdpi.com/article/10.3390/molecules27207068/s1. The ^1^H NMR, ^13^C NMR and IR spectra of novel compounds.

## Sample Availability

Samples of the compounds are not available from the authors, but can be specially prepared upon prior request.
