# Supplementary material for: Synthesis of Trifluoromethylated Monoterpene Amino Alcohols
Source: Molecules. 2022 Oct 20;27(20):7068. doi: 10.3390/molecules27207068 (PMC9607099; doi:10.3390/molecules27207068)
Supplement: Supplementary file 1 [file molecules-27-07068-s001.zip › molecules-1993646-supplementary.pdf]

Supplementary Materials

Molecules

Synthesis of Trifluoromethylated Monoterpene Amino Alcohols

Polina A. Petrova [1], Denis V. Sudarikov [1,2,*], Larisa L. Frolova [1], Roman V. Rumyantcev [3], Svetlana A. Rubtsova [1] and Aleksandr V. Kutchin [1]

[1] Institute of Chemistry, FRC "Komi Scientific Centre", Ural Branch of the Russian Academy of Sciences Pervomayskaya St. 48, 167000 Syktyvkar, Russia

[2] Biologically Active Terpenoids Laboratory, Kazan Federal University, 18 Kremlevskaya Street, 420008 Kazan, Russia

[3] G.A. Razuvaev Institute of Organometallic Chemistry of Russian Academy of Sciences, 49 Tropinina St., 603950 Nizhny Novgorod, Russia

* Correspondence: sudarikov-dv@chemi.komisc.ru

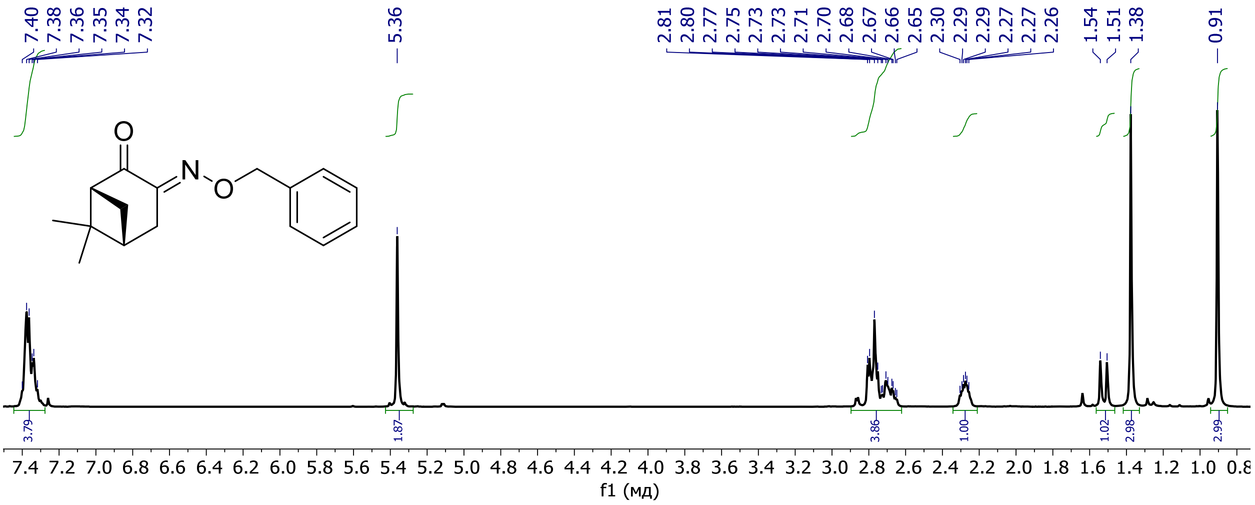
7.40 7.38 7.36 7.35 7.34 7.32
5.36
2.81 2.80 2.77 2.75 2.73 2.73 2.71 2.70 2.68 2.67 2.66 2.65 2.30 2.29 2.29 2.27 2.27 2.26
1.54 1.51 1.38
0.91
O
N
O
3.79
1.87
3.86
1.00
1.02
2.98
2.99
7.4 7.2 7.0 6.8 6.6 6.4 6.2 6.0 5.8 5.6 5.4 5.2 5.0 4.8 4.6 4.4 4.2 4.0 3.8 3.6 3.4 3.2 3.0 2.8 2.6 2.4 2.2 2.0 1.8 1.6 1.4 1.2 1.0 0.8
f1 (мд)

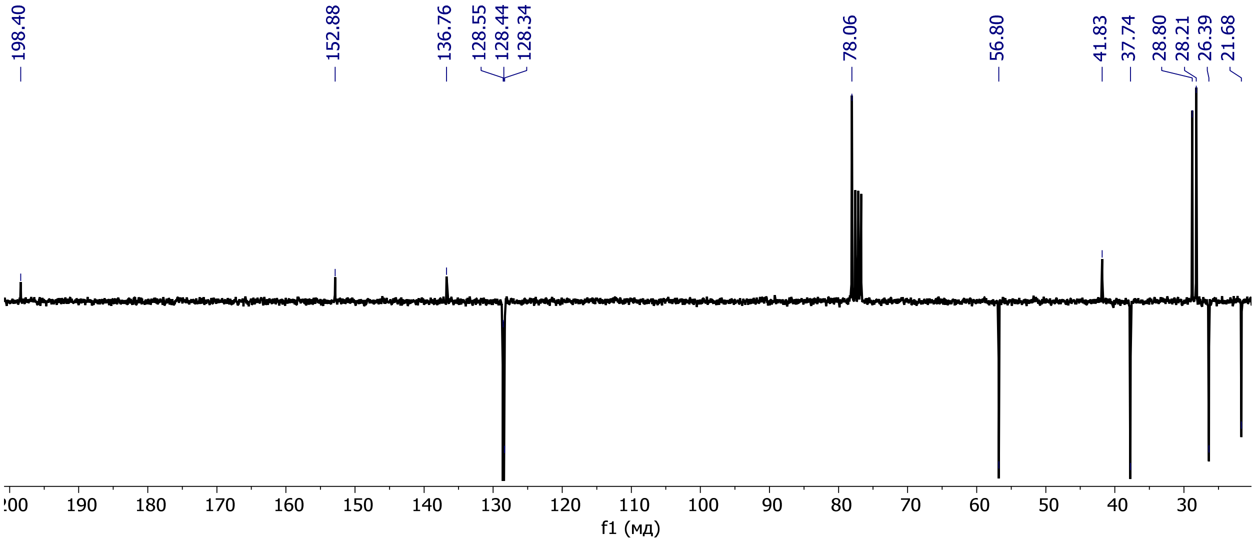
198.40
152.88
136.76
128.55 128.44 128.34
78.06
56.80
41.83
37.74
28.80 28.21 26.39 21.68
200 190 180 170 160 150 140 130 120 110 100 90 80 70 60 50 40 30
f1 (мд)

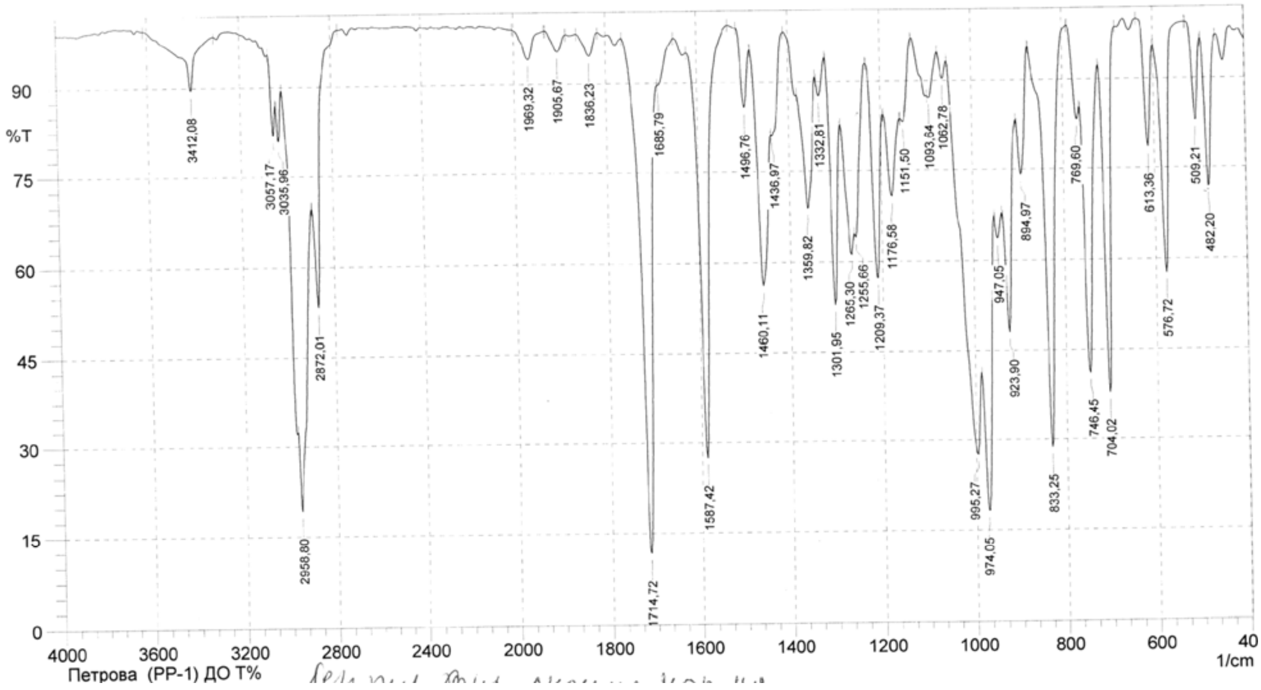
90
%T
75
60
45
30
15
0
3412,08
3057,17
3035,96
2958,80
2872,01
1969,32
1905,67
1836,23
1714,72
1685,79
1587,42
1496,76
1460,11
1436,97
1359,82
1332,81
1301,95
1265,30
1255,66
1209,37
1176,58
1151,50
1093,54
1082,78
995,27
974,05
947,05
923,90
894,97
833,25
769,60
746,45
704,02
613,36
576,72
509,21
482,20
4000 3600 3200 2800 2400 2000 1800 1600 1400 1200 1000 800 600 400
1/cm
Петрова (PP-1) ДО T%

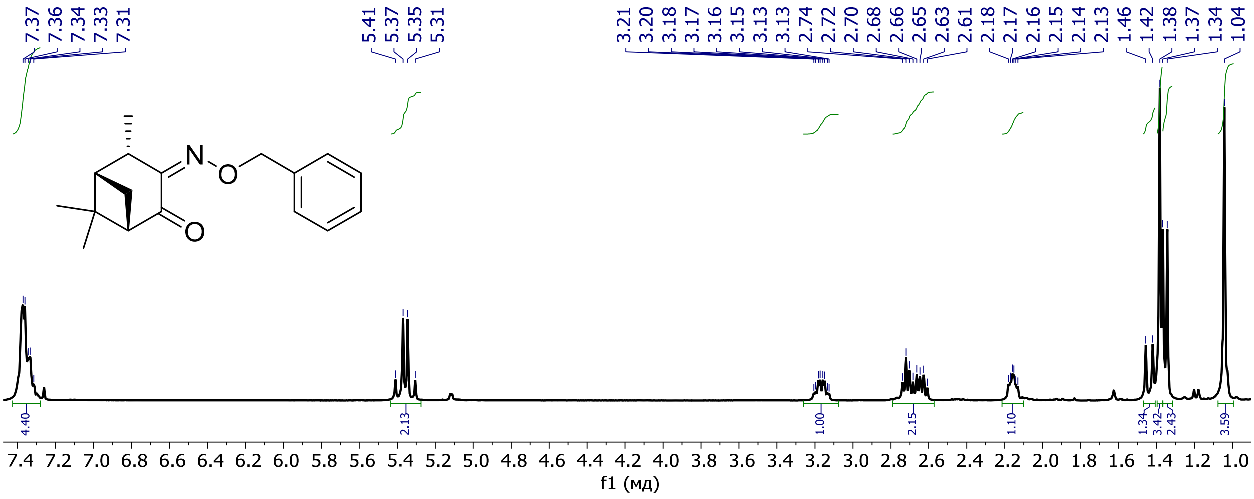
7.37 7.36 7.34 7.33 7.31
5.41 5.37 5.35 5.31
3.21 3.20 3.18 3.17 3.16 3.15 3.13 3.13 2.74 2.72 2.70 2.68 2.66 2.65 2.63 2.61 2.18 2.17 2.16 2.15 2.14 2.13 1.46 1.42 1.38 1.37 1.34 1.04
4.40 2.13 1.00 2.15 1.10 1.34 3.42 2.43 3.59
N O O
7.4 7.2 7.0 6.8 6.6 6.4 6.2 6.0 5.8 5.6 5.4 5.2 5.0 4.8 4.6 4.4 4.2 4.0 3.8 3.6 3.4 3.2 3.0 2.8 2.6 2.4 2.2 2.0 1.8 1.6 1.4 1.2 1.0
f1 (мд)

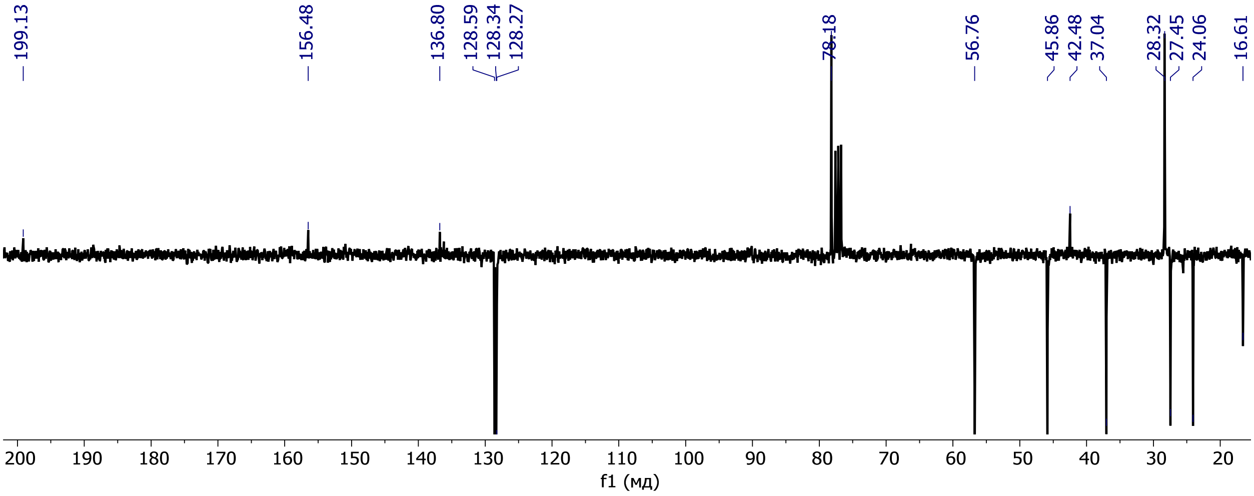
199.13
156.48
136.80 128.59 128.34 128.27
78.18
56.76
45.86 42.48 37.04
28.32 27.45 24.06
16.61
200 190 180 170 160 150 140 130 120 110 100 90 80 70 60 50 40 30 20
f1 (мд)

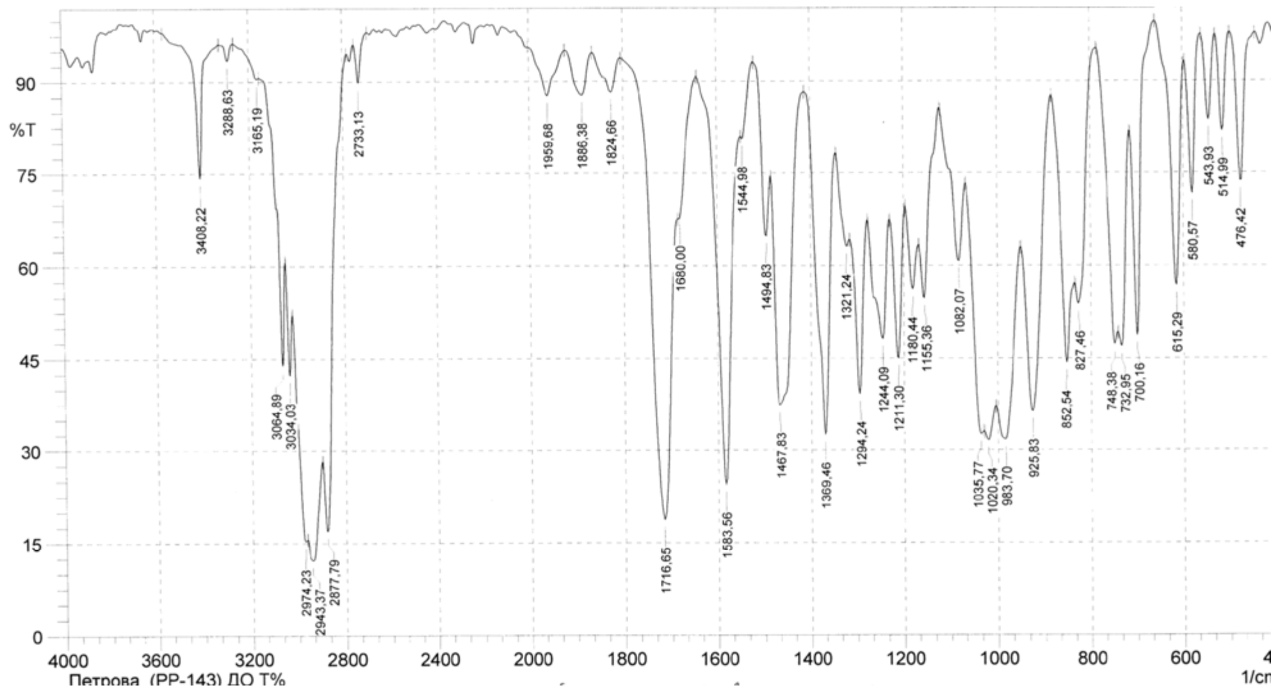
%T
90 75 60 45 30 15 0
3408,22 3288,63 3165,19 3064,89 3034,03 2974,23 2943,37 2877,79 2733,13
1959,68 1886,38 1824,66 1716,65 1680,00 1583,56 1544,98 1494,83 1467,83 1369,46 1321,24 1294,24 1244,09 1211,30 1180,44 1155,36 1082,07 1035,77 1020,34 983,70 925,83 852,54 827,46 748,38 732,95 700,16 615,29 580,57 543,93 514,99 476,42
4000 3600 3200 2800 2400 2000 1800 1600 1400 1200 1000 800 600
1/cm
Петрова (PP-143) ДО Т%

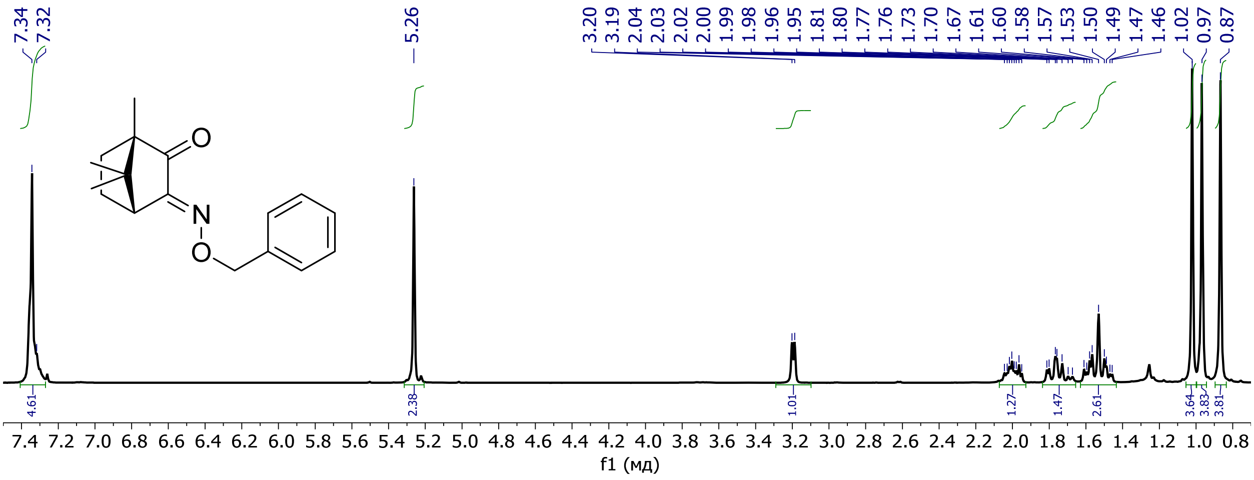
7.34 7.32
5.26
3.20 3.19 2.04 2.03 2.02 2.00 1.99 1.98 1.96 1.95 1.81 1.80 1.77 1.76 1.73 1.70 1.67 1.61 1.60 1.58 1.57 1.53 1.50 1.49 1.47 1.46 1.02 0.97 0.87
O
N
O
4.61 2.38 1.01 1.27 1.47 2.61 3.64 3.83 3.81
7.4 7.2 7.0 6.8 6.6 6.4 6.2 6.0 5.8 5.6 5.4 5.2 5.0 4.8 4.6 4.4 4.2 4.0 3.8 3.6 3.4 3.2 3.0 2.8 2.6 2.4 2.2 2.0 1.8 1.6 1.4 1.2 1.0 0.8
f1 (мд)

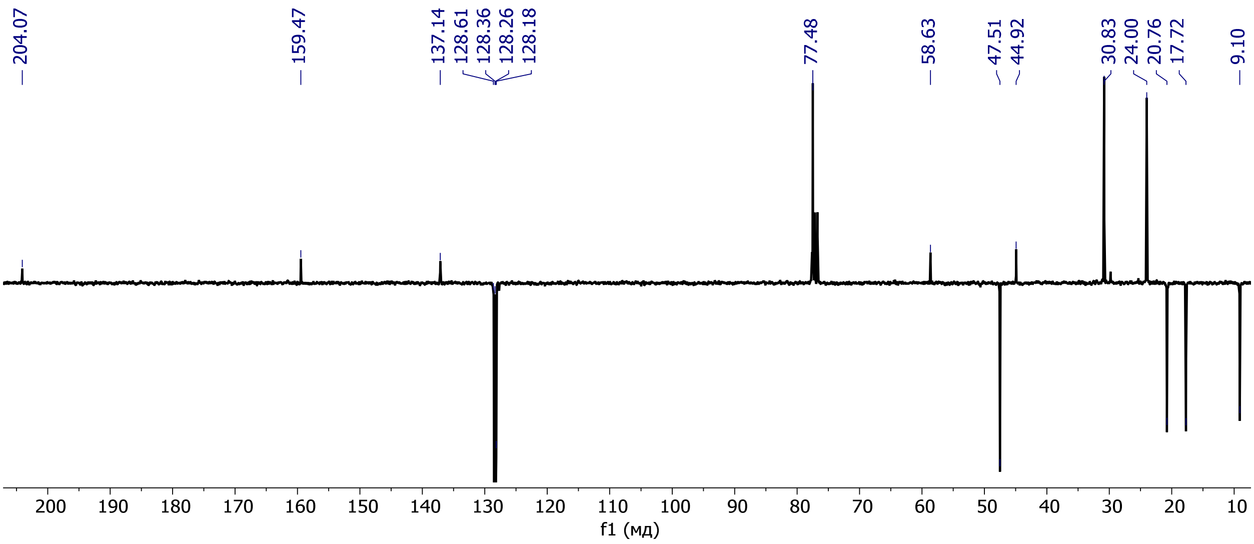
204.07
159.47
137.14 128.61 128.36 128.26 128.18
77.48
58.63
47.51 44.92
30.83 24.00 20.76 17.72
9.10
200 190 180 170 160 150 140 130 120 110 100 90 80 70 60 50 40 30 20 10
f1 (мд)

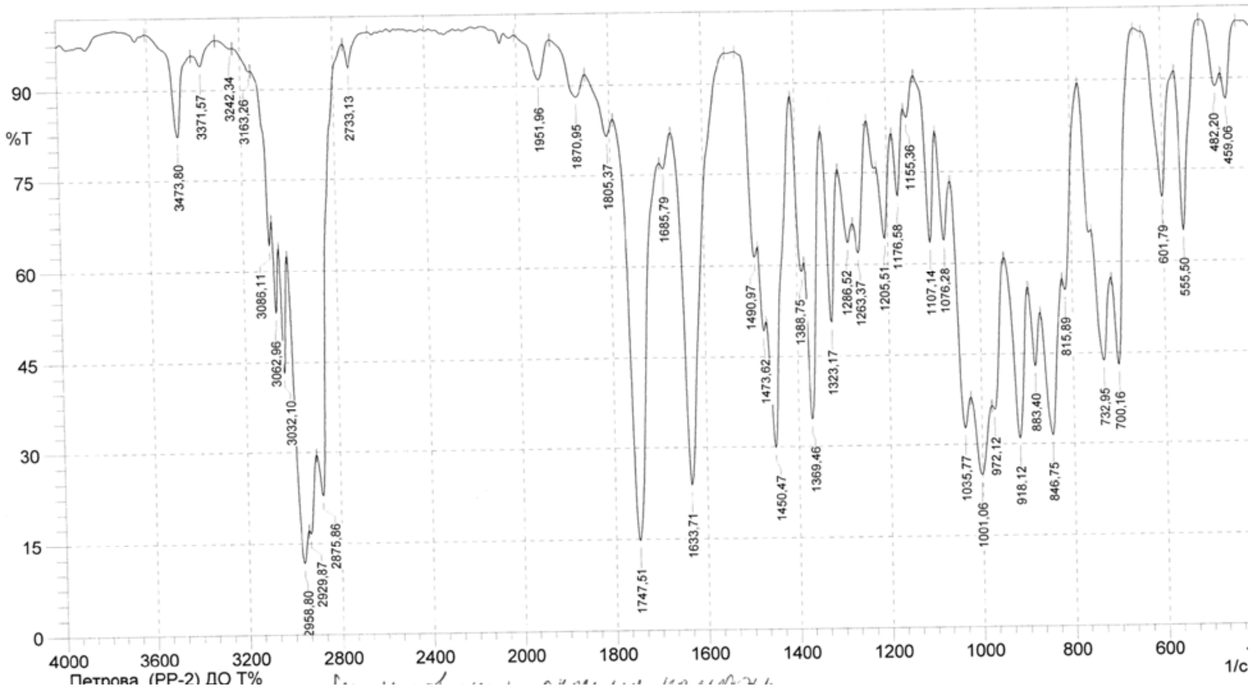
90
%T
75
60
45
30
15
0
3473,80 3371,57 3242,34 3163,26 3086,11 3062,96 3032,10 2958,80 2929,87 2875,86 2733,13 1951,96 1870,95 1805,37 1747,51 1685,79 1633,71 1490,97 1473,62 1450,47 1388,75 1369,46 1323,17 1266,52 1263,37 1205,51 1176,58 1155,36 1107,14 1076,28 1035,77 1001,06 972,12 918,12 883,40 846,75 815,89 732,95 700,16 601,79 555,50 482,20 459,06
4000 3600 3200 2800 2400 2000 1800 1600 1400 1200 1000 800 600
1/c
Петрова (PP-2) ДО T%

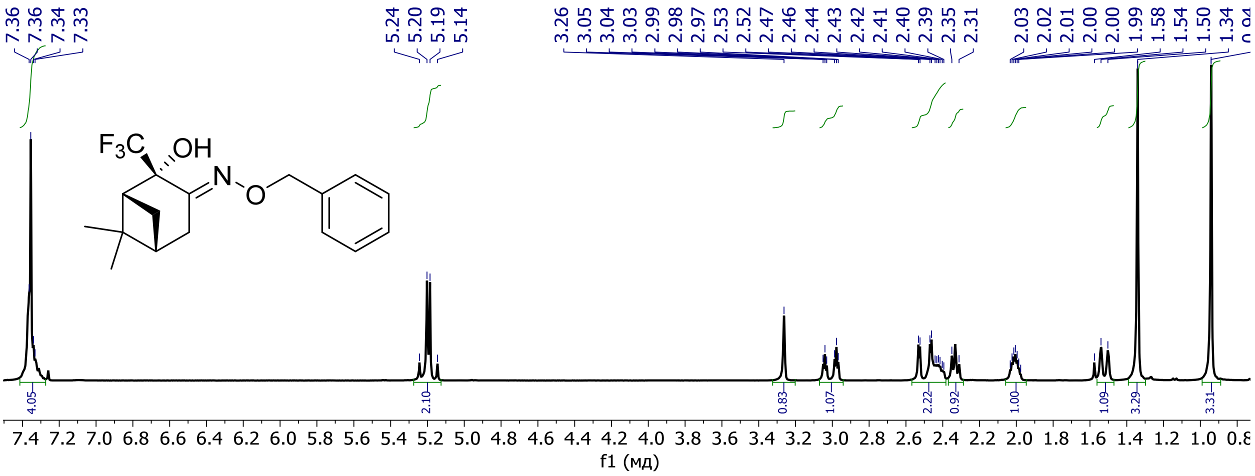
7.36
7.36
7.34
7.33
5.24
5.20
5.19
5.14
3.26
3.05
3.04
3.03
2.99
2.98
2.97
2.53
2.52
2.47
2.46
2.44
2.43
2.42
2.41
2.40
2.39
2.35
2.31
2.03
2.02
2.01
2.00
2.00
1.99
1.58
1.54
1.50
1.34
F_3C
OH
N
O
4.05
2.10
0.83
1.07
2.22
0.92
1.00
1.09
3.29
3.31
7.4 7.2 7.0 6.8 6.6 6.4 6.2 6.0 5.8 5.6 5.4 5.2 5.0 4.8 4.6 4.4 4.2 4.0 3.8 3.6 3.4 3.2 3.0 2.8 2.6 2.4 2.2 2.0 1.8 1.6 1.4 1.2 1.0
f1 (мд)

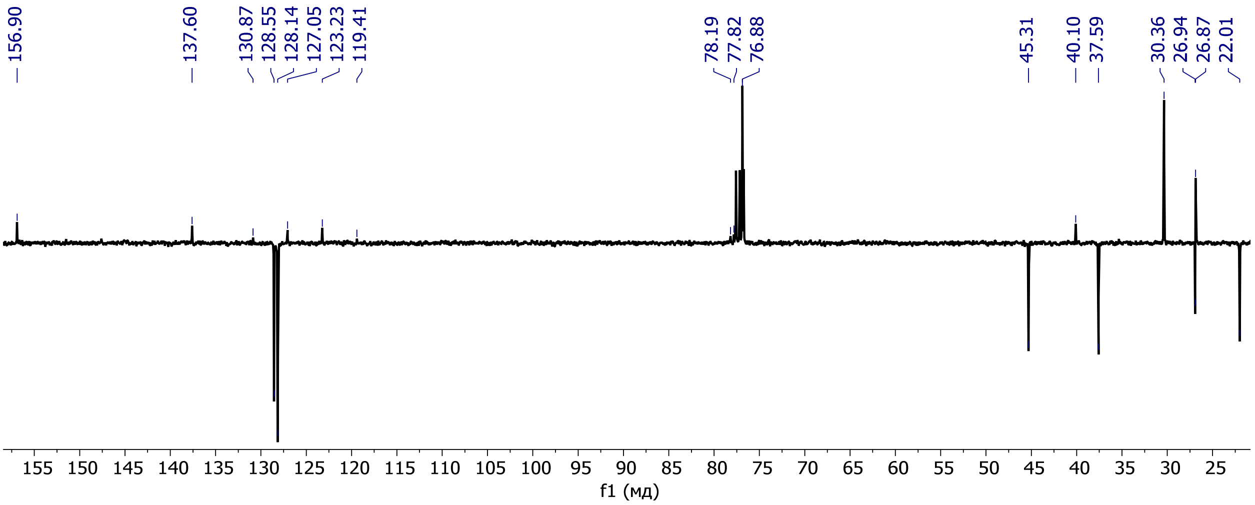
156.90
137.60
130.87
128.55
128.14
127.05
123.23
119.41
78.19
77.82
76.88
45.31
40.10
37.59
30.36
26.94
26.87
22.01
155 150 145 140 135 130 125 120 115 110 105 100 95 90 85 80 75 70 65 60 55 50 45 40 35 30 25
f1 (мд)

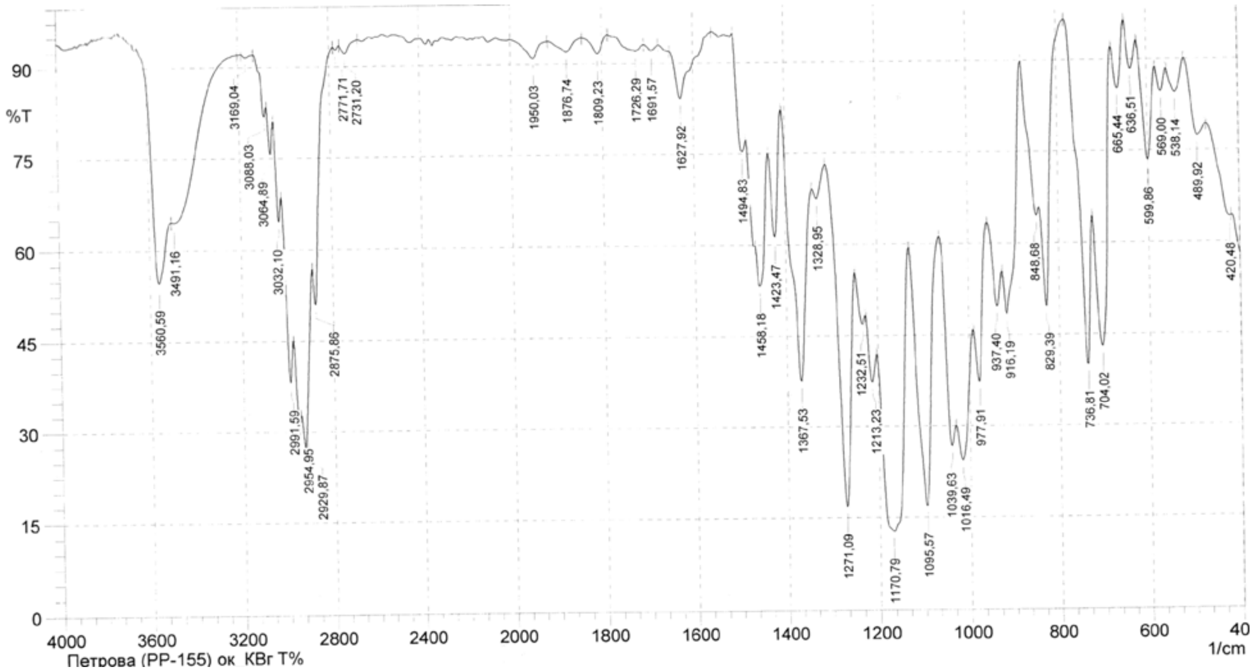
90
%T
75
60
45
30
15
0
3560,59
3491,16
3169,04
3088,03
3064,89
3032,10
2991,59
2954,95
2929,87
2875,86
2771,71
2731,20
1950,03
1876,74
1809,23
1726,29
1691,57
1627,92
1494,83
1458,18
1423,47
1367,53
1328,95
1271,09
1232,51
1213,23
1170,79
1095,57
1039,63
1016,49
977,91
937,40
916,19
848,68
829,39
736,81
704,02
665,44
636,51
599,86
569,00
538,14
489,92
420,48
4000 3600 3200 2800 2400 2000 1800 1600 1400 1200 1000 800 600 40
1/cm
Петрова (PP-155) ок KBr T%

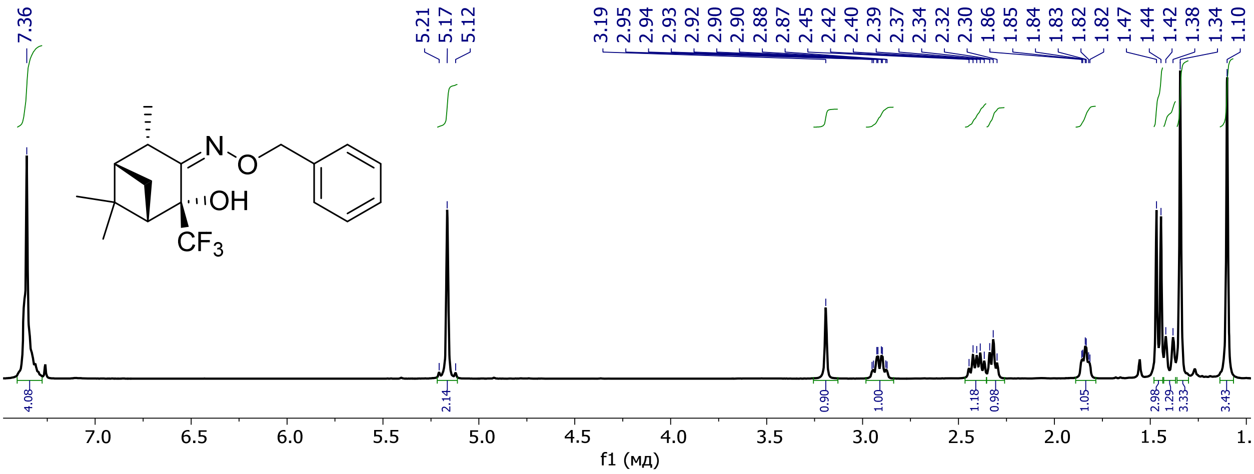
7.36
5.21
5.17
5.12
3.19
2.95
2.94
2.93
2.92
2.90
2.90
2.88
2.87
2.45
2.42
2.40
2.39
2.37
2.34
2.32
2.30
1.86
1.85
1.84
1.83
1.82
1.82
1.47
1.44
1.42
1.38
1.34
1.10
N
O
OH
CF3
4.08
2.14
0.90
1.00
1.18
0.98
1.05
2.98
1.29
3.33
3.43
7.0 6.5 6.0 5.5 5.0 4.5 4.0 3.5 3.0 2.5 2.0 1.5
f1 (мд)

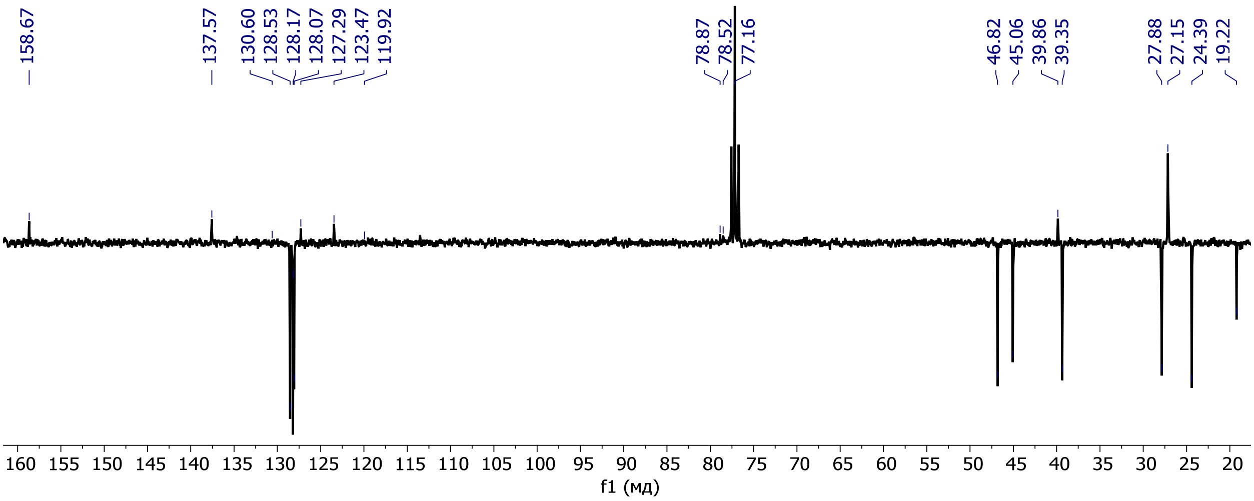
158.67
137.57
130.60
128.53
128.17
128.07
127.29
123.47
119.92
78.87
78.52
77.16
46.82
45.06
39.86
39.35
27.88
27.15
24.39
19.22
160 155 150 145 140 135 130 125 120 115 110 105 100 95 90 85 80 75 70 65 60 55 50 45 40 35 30 25 20
f1 (мд)

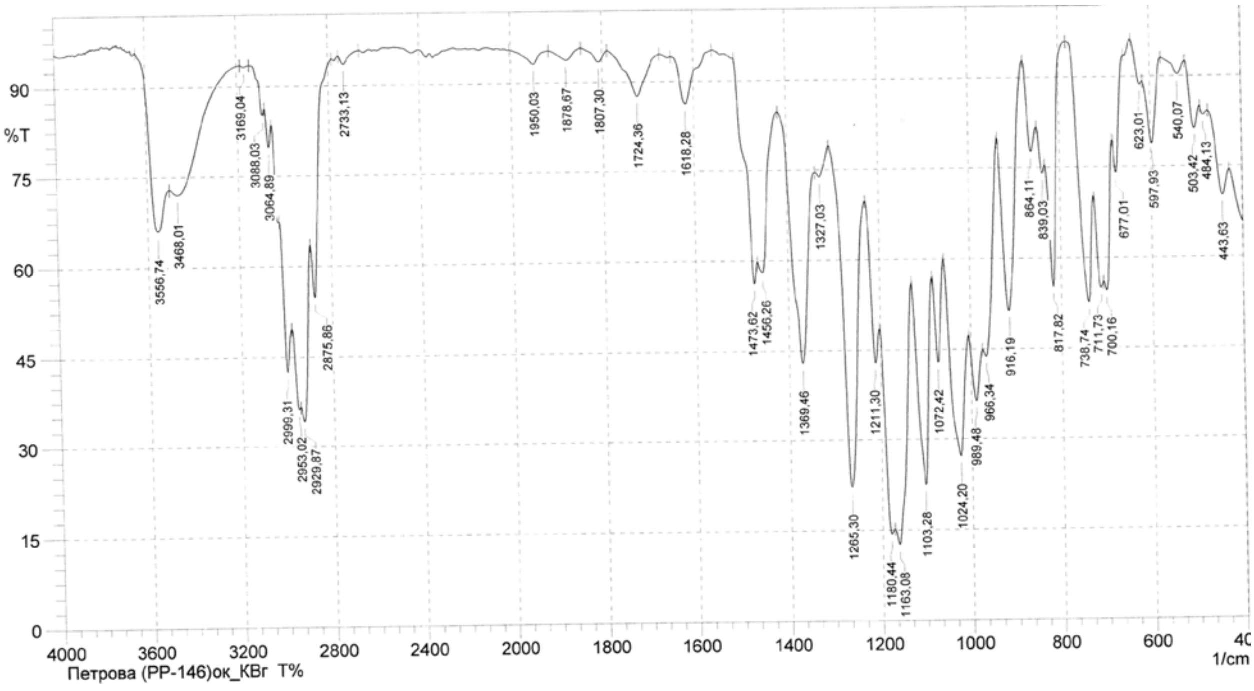
%T
90
75
60
45
30
15
0
3556,74
3468,01
3169,04
3088,03
3064,89
2999,31
2953,02
2929,87
2875,86
2733,13
1950,03
1878,67
1807,30
1724,36
1618,28
1473,62
1456,26
1369,46
1327,03
1265,30
1211,30
1180,44
1163,08
1103,28
1072,42
1024,20
989,48
966,34
916,19
864,11
839,03
817,82
738,74
711,73
700,16
677,01
623,01
597,93
540,07
503,42
484,13
443,63
4000 3600 3200 2800 2400 2000 1800 1600 1400 1200 1000 800 600
1/cm
Петрова (PP-146)ок_KBr T%

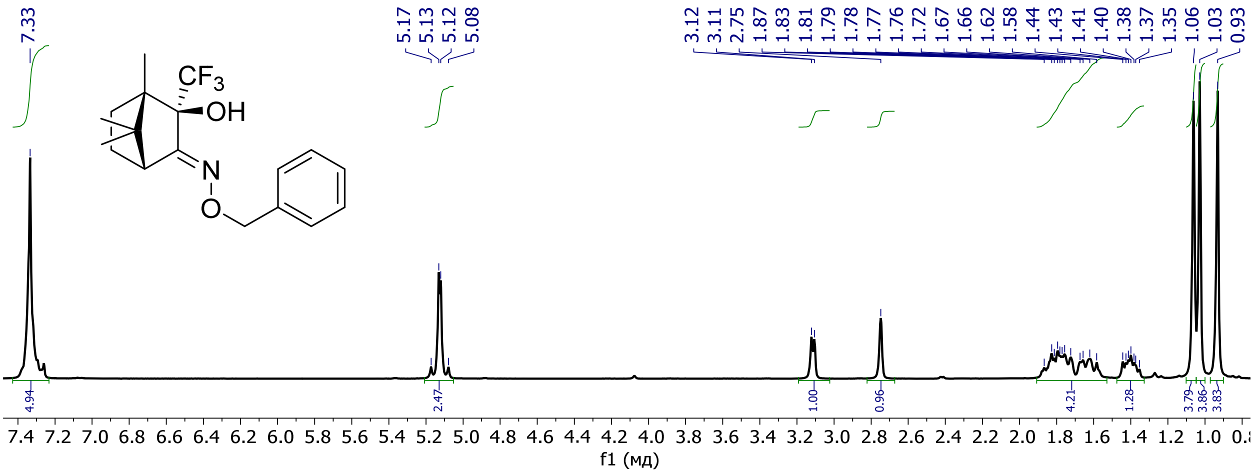
7.33
5.17 5.13 5.12 5.08
3.12 3.11 2.75 1.87 1.83 1.81 1.79 1.78 1.77 1.76 1.72 1.67 1.66 1.62 1.58 1.44 1.43 1.41 1.40 1.38 1.37 1.35 1.06 1.03 0.93
CF3
OH
N
O
4.94
2.47
1.00
0.96
4.21
1.28
3.79
3.86
3.83
f1 (мд)

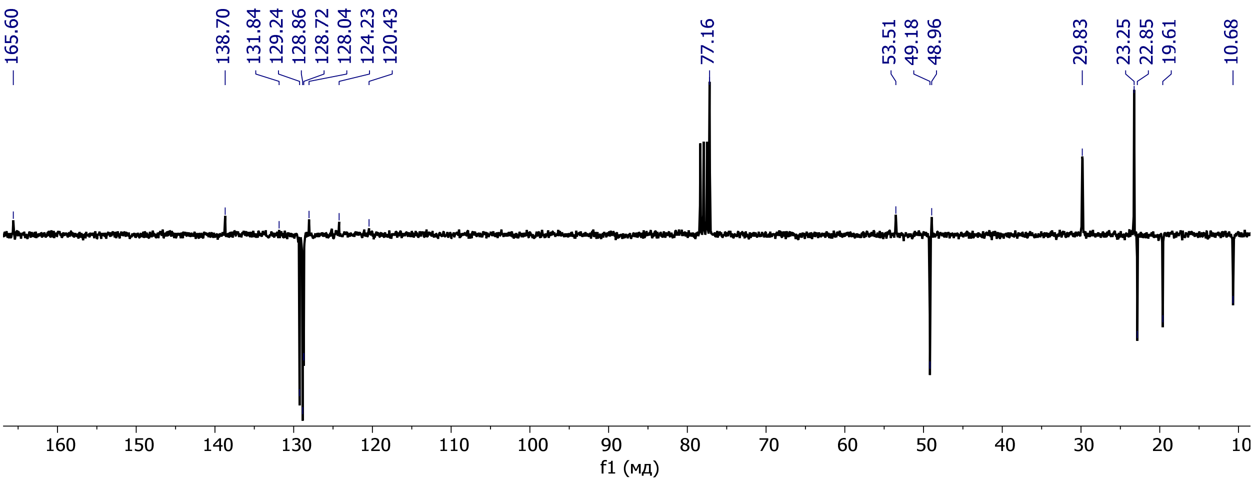
165.60
138.70 131.84 129.24 128.86 128.72 128.04 124.23 120.43
77.16
53.51 49.18 48.96
29.83
23.25 22.85 19.61
10.68
f1 (мд)

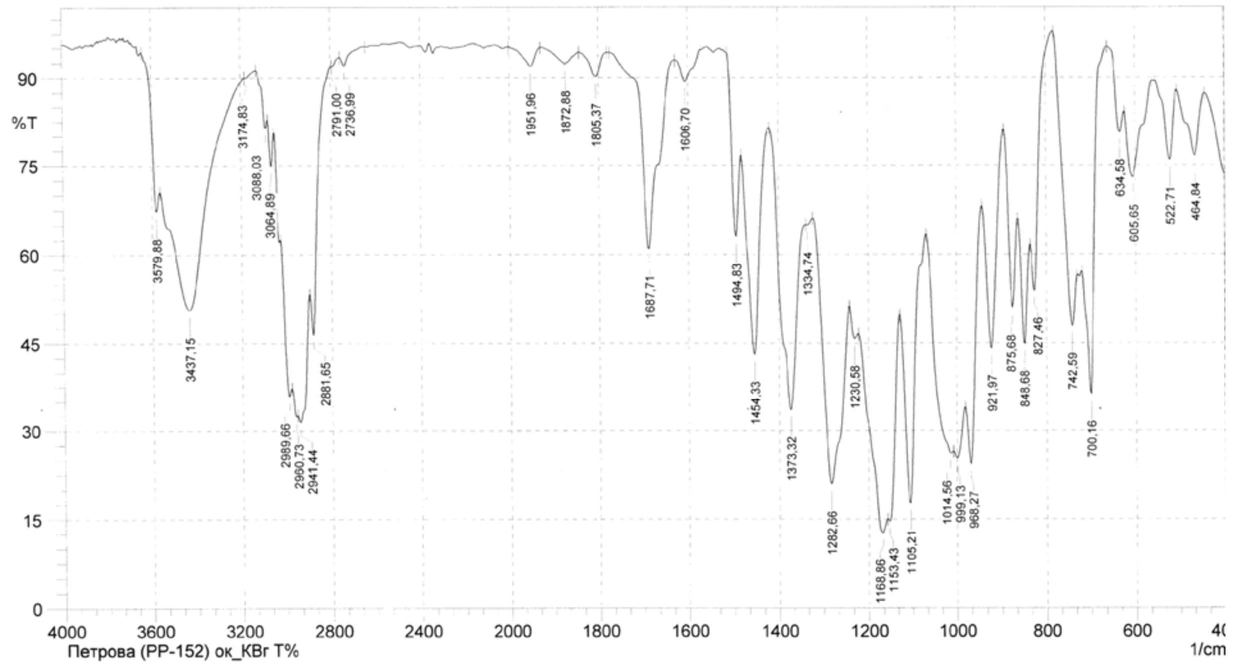
%T
3579.88
3437.15
3174.83
3088.03
3064.89
2989.66
2960.73
2941.44
2881.65
2791.00
2736.99
1951.96
1872.88
1805.37
1687.71
1606.70
1494.83
1454.33
1373.32
1334.74
1282.66
1230.58
1168.86
1153.43
1105.21
1014.56
999.13
968.27
921.97
875.68
848.68
827.46
742.59
700.16
634.58
605.65
522.71
464.84
1/cm
Петрова (PP-152) ок_KBr T%

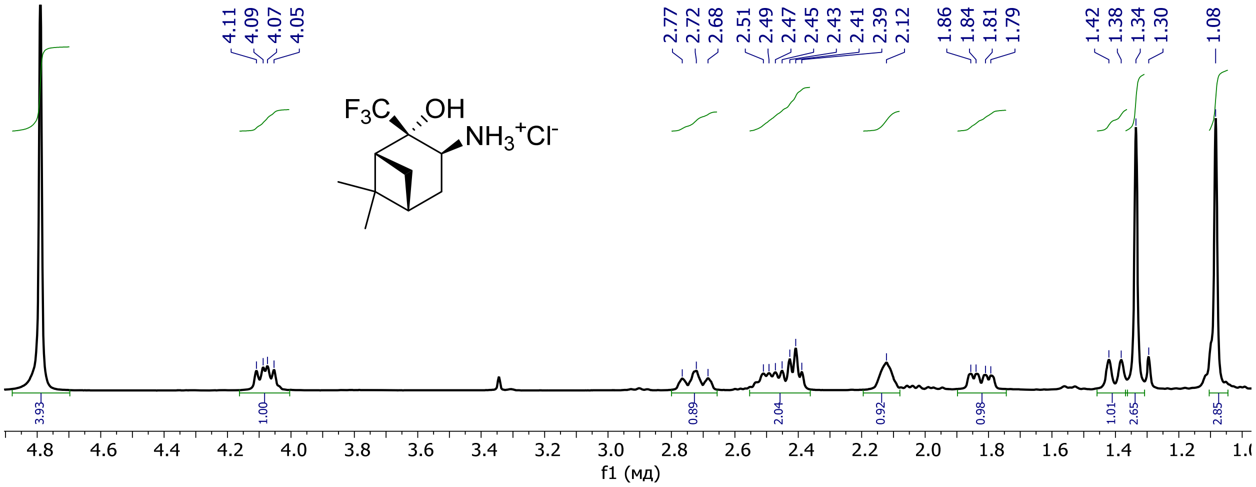
4.11 4.09 4.07 4.05
2.77 2.72 2.68 2.51 2.49 2.47 2.45 2.43 2.41 2.39 2.12
1.86 1.84 1.81 1.79
1.42 1.38 1.34 1.30
1.08
F_3C
OH
$NH_3^+Cl^-$
3.93 1.00 0.89 2.04 0.92 0.98 1.01 2.65 2.85
4.8 4.6 4.4 4.2 4.0 3.8 3.6 3.4 3.2 3.0 2.8 2.6 2.4 2.2 2.0 1.8 1.6 1.4 1.2
f1 (мд)

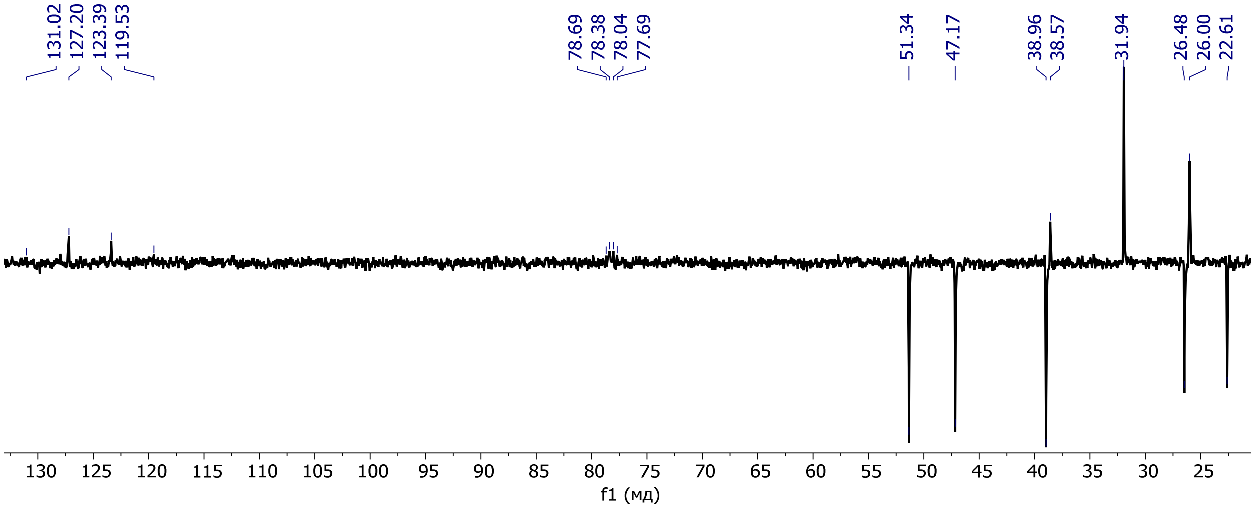
131.02 127.20 123.39 119.53
78.69 78.38 78.04 77.69
51.34
47.17
38.96 38.57
31.94
26.48 26.00 22.61
130 125 120 115 110 105 100 95 90 85 80 75 70 65 60 55 50 45 40 35 30 25
f1 (мд)

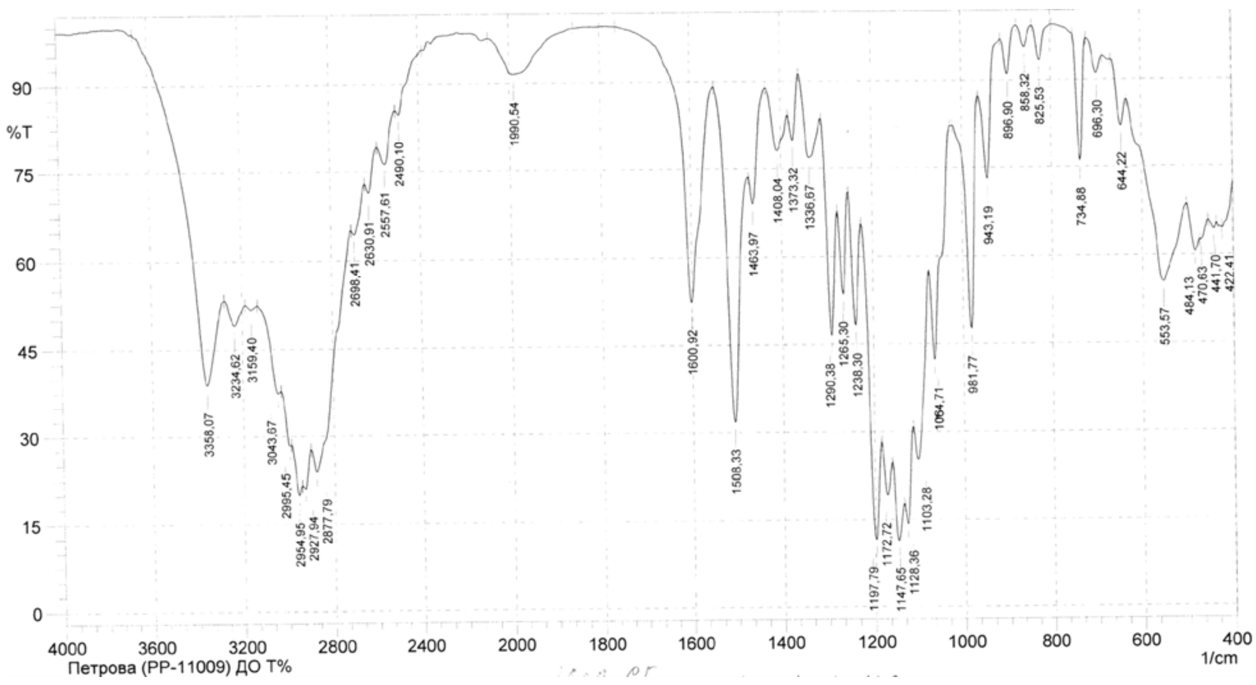
90
%T
75
60
45
30
15
0
3358,07 3234,62 3159,40 3043,67 2995,45 2954,95 2927,94 2877,79 2698,41 2630,91 2557,61 2490,10
1990,54
1600,92 1508,33 1463,97 1408,04 1373,32 1336,67 1290,38 1265,30 1238,30
1197,79 1172,72 1147,65 1128,36 1103,28 1064,71 981,77 943,19
896,90 858,32 825,53 734,88 696,30 644,22 553,57 484,13 470,63 441,70 422,41
4000 3600 3200 2800 2400 2000 1800 1600 1400 1200 1000 800 600 400
1/cm
Петрова (PP-11009) ДО Т%

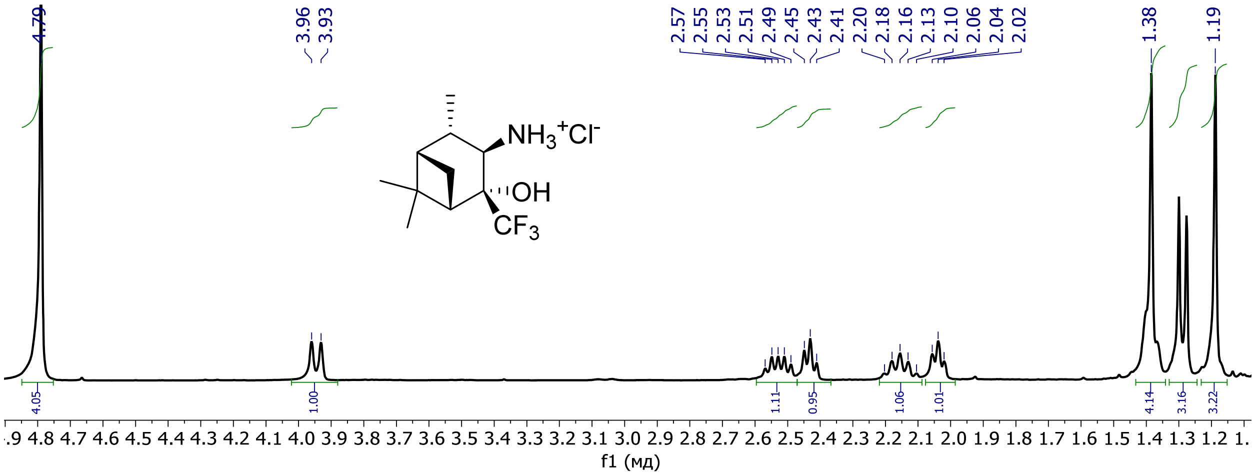
4.79
3.96
3.93
2.57
2.55
2.53
2.51
2.49
2.45
2.43
2.41
2.20
2.18
2.16
2.13
2.10
2.06
2.04
2.02
1.38
1.19
NH3+Cl-
OH
CF3
4.05
1.00
1.11
0.95
1.06
1.01
4.14
3.16
3.22
f1 (мд)

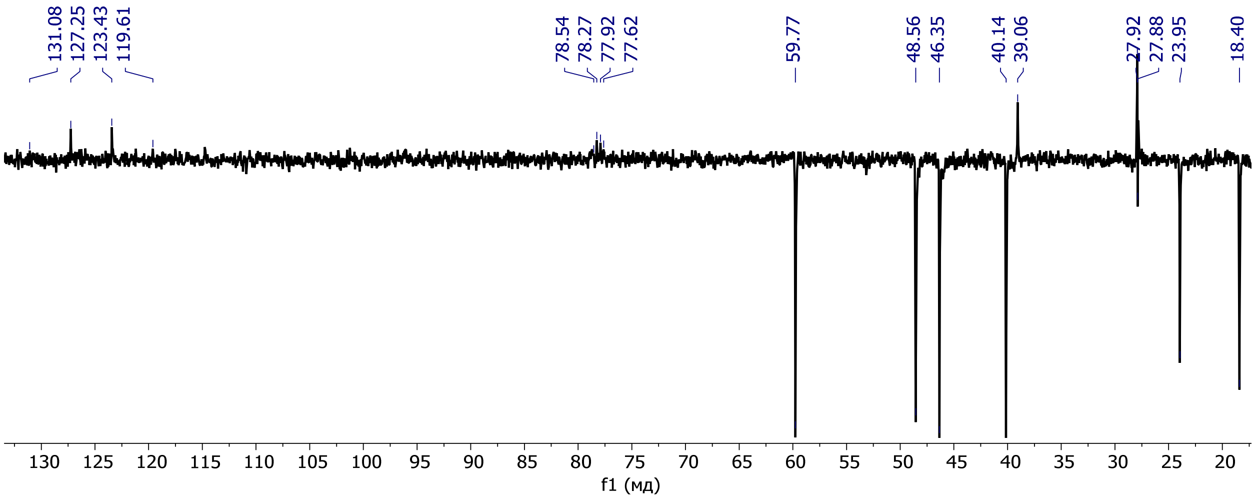
131.08
127.25
123.43
119.61
78.54
78.27
77.92
77.62
59.77
48.56
46.35
40.14
39.06
27.92
27.88
23.95
18.40
f1 (мд)

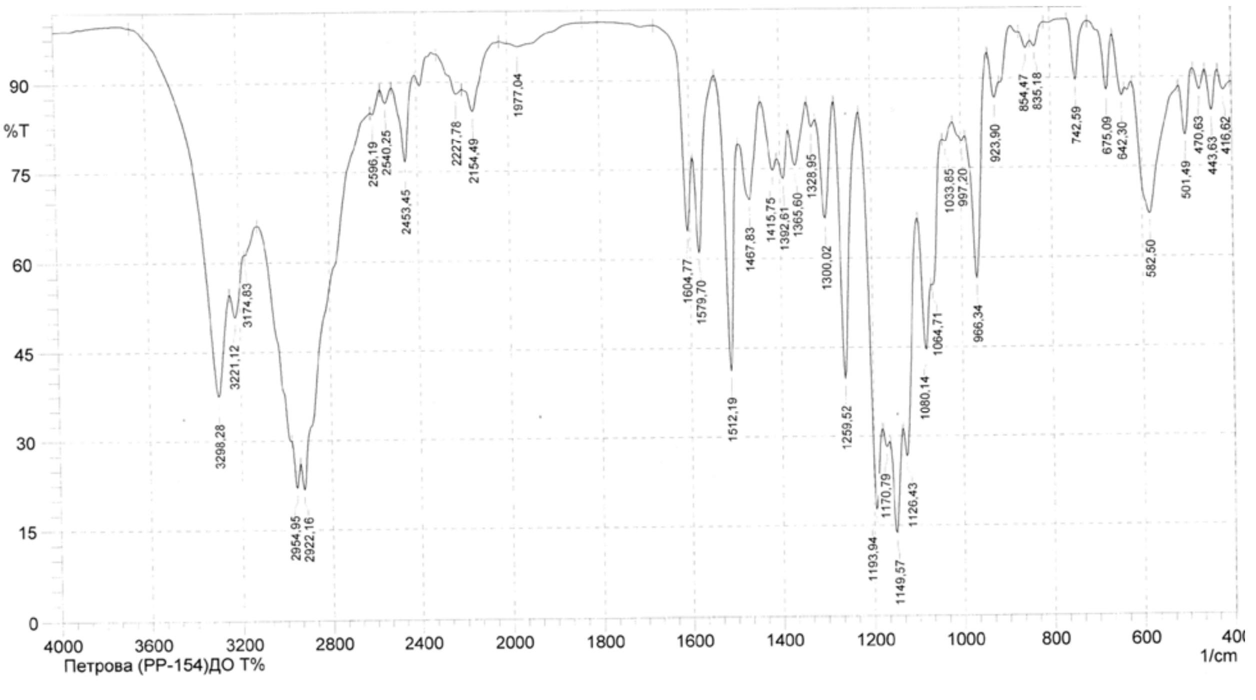
%T
1/cm
Петрова (PP-154)ДО T%
3298,28
3221,12
3174,83
2954,95
2922,16
2596,19
2540,25
2453,45
2227,78
2154,49
1977,04
1604,77
1579,70
1512,19
1467,83
1415,75
1392,61
1365,60
1328,95
1300,02
1259,52
1193,94
1170,79
1149,57
1126,43
1080,14
1064,71
1033,85
997,20
966,34
923,90
854,47
835,18
742,59
675,09
642,30
582,50
501,49
470,63
443,63
416,62

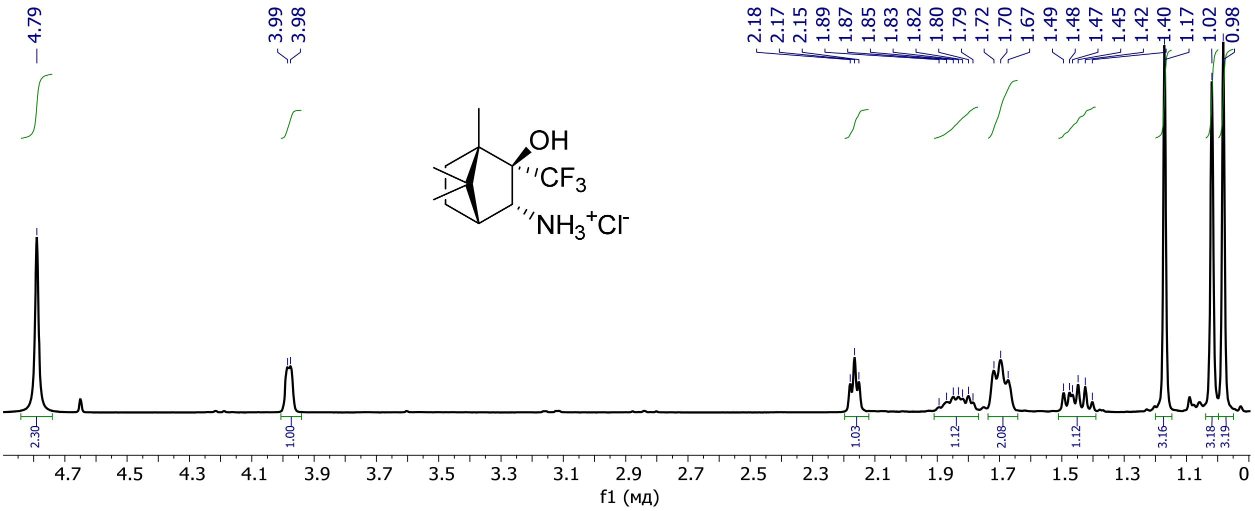
4.79
3.99
3.98
2.18 2.17 2.15 1.89 1.87 1.85 1.83 1.82 1.80 1.79 1.72 1.70 1.67 1.49 1.48 1.47 1.45 1.42 1.40 1.17 1.02 0.98
OH
CF3
NH3+Cl-
2.30 1.00 1.03 1.12 2.08 1.12 3.16 3.18 3.19
f1 (мд)

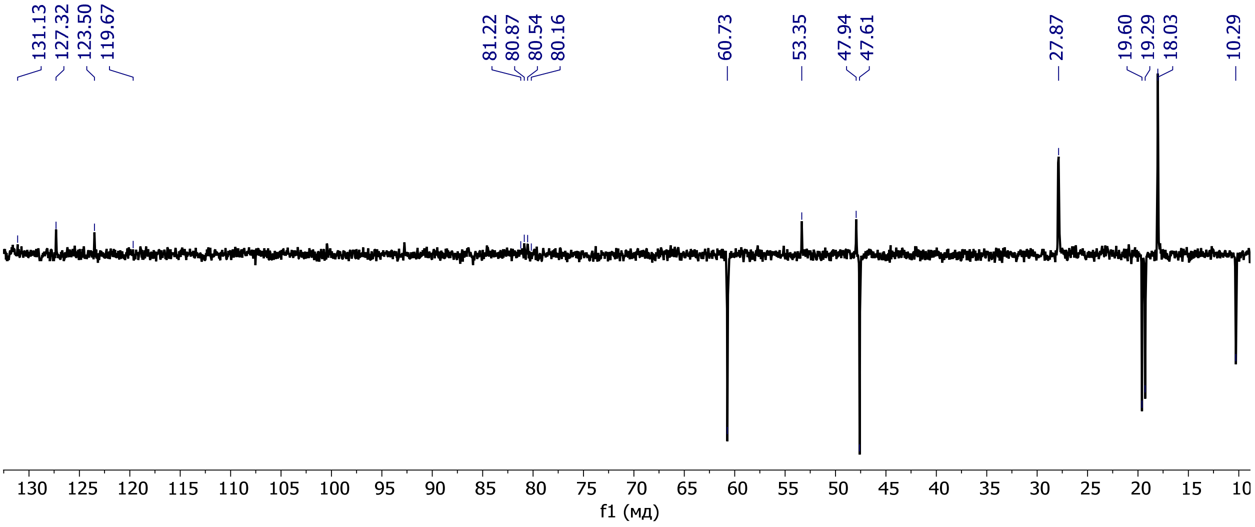
131.13 127.32 123.50 119.67
81.22 80.87 80.54 80.16
60.73
53.35
47.94 47.61
27.87
19.60 19.29 18.03
10.29
f1 (мд)

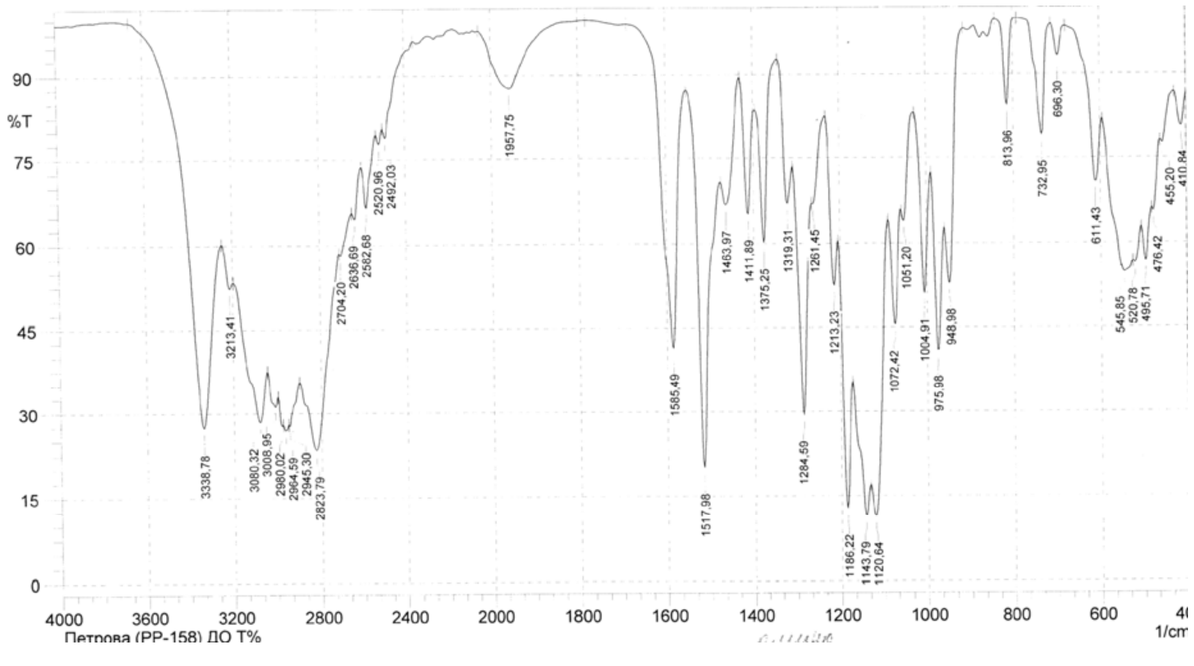
%T
3338,78 3213,41 3080,32 3008,95 2980,02 2964,59 2945,30 2823,79 2704,20 2636,69 2582,68 2520,96 2492,03 1957,75 1585,49 1517,98 1463,97 1411,89 1375,25 1319,31 1284,59 1261,45 1213,23 1186,22 1143,79 1120,64 1072,42 1051,20 1004,91 975,98 948,98 813,96 732,95 696,30 611,43 545,85 520,78 495,71 476,42 455,20 410,84
1/cm
Петрова (PP-158) ДО Т%
